# Discovery of VU0467319: an M_1_ Positive Allosteric Modulator Candidate That Advanced into Clinical Trials

**DOI:** 10.1021/acschemneuro.4c00769

**Published:** 2024-12-11

**Authors:** Michael S. Poslunsey, Michael R. Wood, Changho Han, Shaun R. Stauffer, Joseph D. Panarese, Bruce J. Melancon, Julie L. Engers, Jonathan W. Dickerson, Weimin Peng, Meredith J. Noetzel, Hyekyung P. Cho, Alice L. Rodriguez, Corey R. Hopkins, Ryan Morrison, Rachel D. Crouch, Thomas M. Bridges, Anna L. Blobaum, Olivier Boutaud, J. Scott Daniels, Michael J. Kates, Arlindo Castelhano, Jerri M. Rook, Colleen M. Niswender, Carrie K. Jones, P. Jeffrey Conn, Craig W. Lindsley

**Affiliations:** † Warren Center for Neuroscience Drug Discovery, Vanderbilt University, Nashville, Tennessee 37232, United States; ‡ Department of Pharmacology, Vanderbilt University School of Medicine, Nashville, Tennessee 37232, United States; § Department of Chemistry, Vanderbilt University, Nashville Tennessee 37232, United States; ∥ Vanderbilt Kennedy Center, Vanderbilt University Medical Center, Nashville, Tennessee 37232, United States; ⊥ Vanderbilt Brain Institute, Vanderbilt University, Nashville, Tennessee 37232, United States; # Vanderbilt Institute of Chemical Biology, Vanderbilt University, Nashville, Tennessee 37232, United States; ∇ Davos Pharma, Upper Saddle River, New Jersey 07458, United States

**Keywords:** muscarinic acetylcholine receptor subtype 1 (M_1_), positive allosteric modulator (PAM), cognition, metabolism

## Abstract

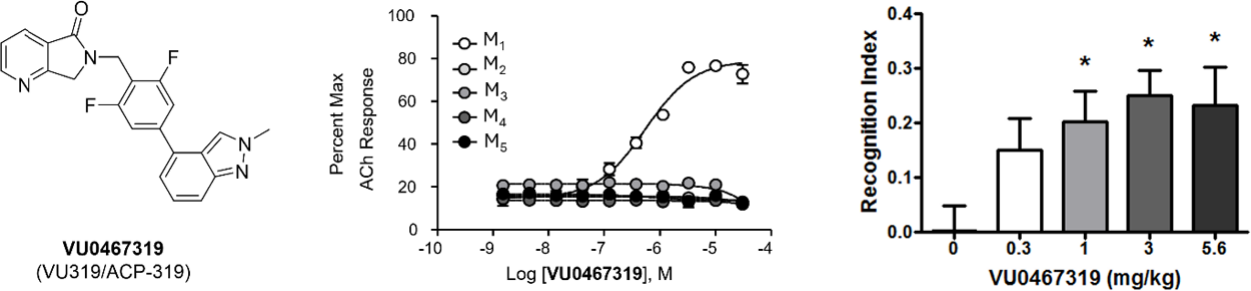

Herein we detail the *first disclosure* of VU0467319 (VU319), an M_1_ Positive Allosteric Modulator (PAM) clinical candidate that successfully completed a Phase I Single Ascending Dose (SAD) clinical trial. VU319 (**16**) is a moderately potent M_1_ PAM (M_1_ PAM EC_50_ = 492 nM ± 2.9 nM, 71.3 ± 9.9% ACh Max), with minimal M_1_ agonism (EC_50_ > 30 μM), that displayed high CNS penetration (*K*_p_*s* > 0.67 and *K*_p,uu_*s* > 0.9) and multispecies pharmacokinetics permissive of further development. Based on robust efficacy in multiple preclinical models of cognition, an ancillary pharmacology profile devoid of appreciable off-target activities, and a lack of cholinergic adverse effects (AEs) in rats, dogs and nonhuman primates, VU319 advanced into IND-enabling studies. After completing 4-week rat and dog GLP toxicology without AEs, including absence of cholinergic effects, the first in human Phase I SAD clinical trial of VU319 (NCT03220295) was performed at Vanderbilt, where a similar lack of adverse effects, including absence of cholinergic effects was noted. Moreover, signals of target engagement were seen at the highest dose tested. Thus, VU319 demonstrated the feasibility of achieving selective targeting of central M_1_ muscarinic receptors without eliciting cholinergic AEs that have plagued other drugs targeting CNS cholinergic neurotransmission.

## Introduction

**Figure 1:**
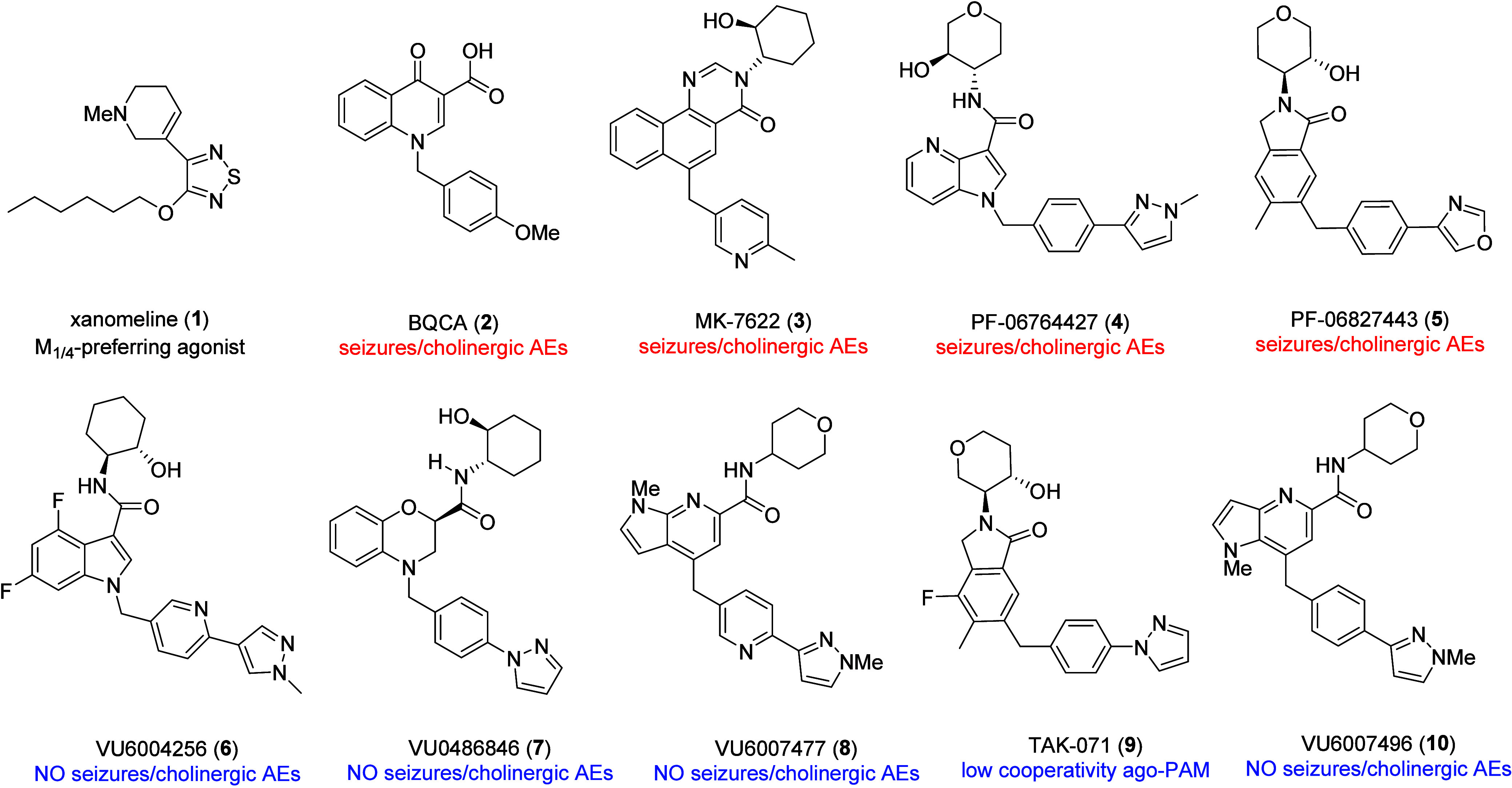
Structures of the M_1_/M_4_-preferring xanomeline (**1**), representative M_1_ PAMs with cholinergic AEs **2**-**5**, and representative M_1_ PAMs with lesser levels of M_1_ agonism **6**-**10**, that showed no cholinergic AEs.

The selective activation of muscarinic acetylcholine receptors (mAChRs or M_1–5_) has been a long sought after goal since clinical trials in the 1980s demonstrated that “M_1_ agonists” had robust efficacy in improving cognition in Alzheimer’s patients; however, these unselective mAChR agonists activated peripheral M_2_ and M_3_ receptors resulting in significant adverse cholinergic events, SLUDGE (salivation, lacrimation, urination, defecation, gastrointestinal distress and emesis).^1−6^ The most successful of these unselective agonists was xanomeline (**1**), an M_1_/M_4_ preferring agonist (Figure 1), that at therapeutic concentrations, activates all the mAChRs. Karuna employed a creative strategy to revive **1** recently, gaining FDA approval of the combination of xanomeline **1** coadministered with the peripherally restricted muscarinic antagonist trospium to reduce SLUDGE effects (combination known as KarXT, cobenfy).^7−9^ While cobenfy represents an important advance, balancing the degree of mAChR activation and inhibition can lead to adverse effects across heterogeneous patient populations. As a chronic, daily maintenance therapeutic, a highly selective M_1_ activator, devoid of the need for coadministration of a peripheral mAChR antagonist may offer a more attractive option for many patients.^10^

Over 20 years ago, the concept of allosteric modulation of GPCRs was exploited to provide highly selective positive allosteric modulators (PAMs) of both Family A and C GPCRs,^11,12^ including the first M_1_ PAM, BQCA (**2**) by Merck.^13−15^ Since those initial discoveries, our laboratories and others have been developing M_1_ PAMs with a diverse array of pharmacological profiles (Figure 1). Clinical data, genetic data and preclinical data with tools **1**-**10** strongly suggests M_1_ PAMs can treat cognitive dysfunction in schizophrenia, Alzheimer’s disease, prion diseases and other CNS disorders.^14−26^ However, caveats appeared and demonstrated that all M_1_ PAMs are not equivalent; moreover, intrinsic agonism, signal bias and the degree of cooperativity may have been key factors in adverse events, cholinergic side effects and candidate advancement into the clinic.^14−26^ Based on these caveats, and data reported with M_1_ PAMs from prior programs, we took steps to derisk our M_1_ PAM chemotypes, both preclinically and clinically.

Here, we detail for the first time the discovery and development of VU319, a novel M_1_ PAM, minimal agonism, that displayed robust pro-cognitive efficacy in preclinical models and was devoid of SLUDGE effects in mice, rats, dogs and nonhuman primates. In a Phase I SAD clinical trial of VU319 (NCT03220295),^27−30^ SLUDGE effects were not observed, and signs of precognitive efficacy were evident. Herein, we describe the discovery of VU319, its in vitro and in vivo pharmacological and DMPK profile, its safety/toxicology in IND-enabling studies and its progression into human testing.

## Results and Discussion

### Design

**Figure 2:**
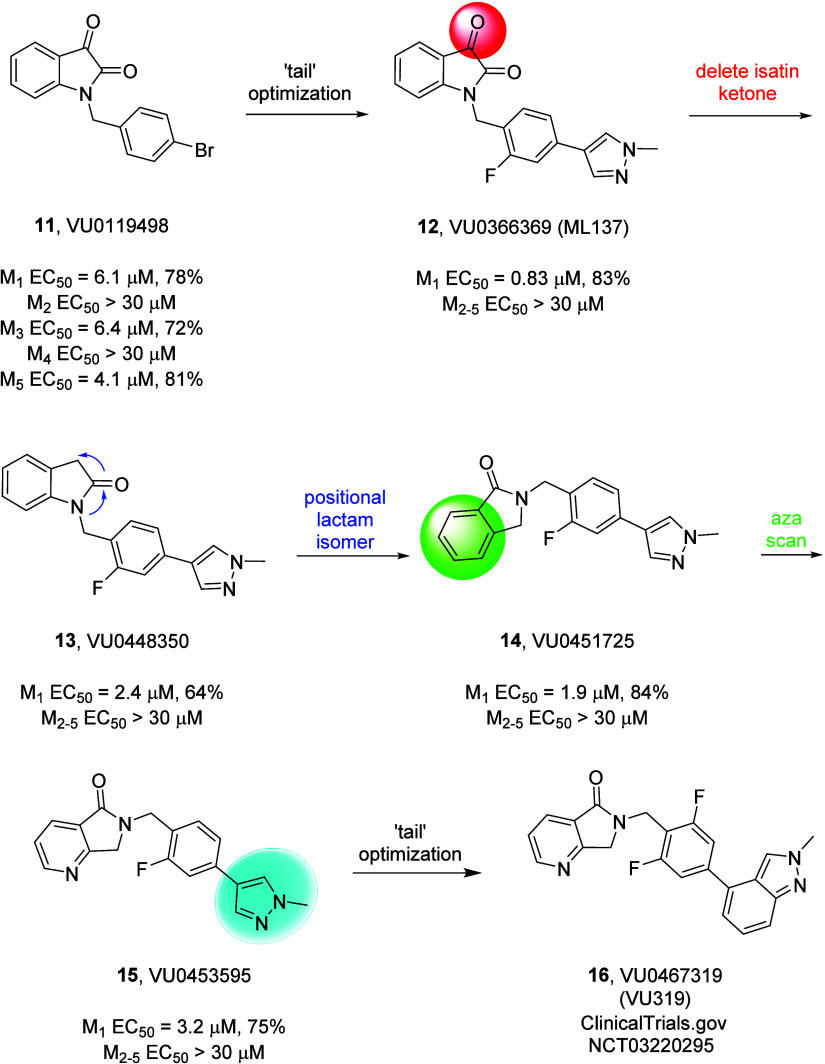
Roadmap to the discovery of VU0467319 (VU319, **16**). From an unselective, *pan*-G_q_-mAChR (M_1_, M_3_, M_5_) PAM **11**, harboring an electrophilic and reactive isatin moiety, to a highly selective M_1_ PAM clinical candidate, VU319 (**16**) with minimal agonism and no reactive functionality.

Results with BQCA (**2**), a potent M_1_ PAM with high intrinsic M_1_ agonist activity, indicated that this pharmacological profile may be undesirable, and that an M_1_ PAM with lower levels of intrinsic M_1_ agonism might be better tolerated an cause fewer cholinergic adverse evenets.^13−15,17^ A functional M_1_ high-throughput screen at Vanderbilt identified BQCA-like PAMs, and a single chemotype that displayed no M_1_ agonism up to 30 μM, a simple isatin derivative, VU0119498 (**11**).^31^ However, **11** proved to be a PAM of all three G_q_-coupled mAChRs (M_1_, M_3_ and M_5_) but inactive on the G_i/o_-coupled mAChRs (M_2_ and M_4_); moreover, **11** harbored an electrophilic and reactive isatin moiety, rendering it unattractive as a lead for an M_1_ PAM development effort.^31^ However, with only a single hit lacking appreciable M_1_ agonism, the medicinal chemists had no choice but to attempt to remove the undesirable isatin moiety of **11** while developing SAR to engender selective M_1_ PAM pharmacology with minimal M_1_ agonism. As previously described, optimization efforts on the southern benzyl tail identified modifications that abolished M_3_ and M_5_ PAM activity, affording a selective M_1_ PAM, ML137 (**12**), with minimal agonism, yet still possessing the isatin core.^32^ Deletion of the ketone moiety in **12** afforded lactam **13** (VU0448350), a weak but selective M_1_ PAM, again with minimal M_1_ agonism.^33^ Surveying alternative positional lactam isomers led to the discovery of VU0451725 (**14**), a selective M_1_ PAM with minimal agonism. An aza scan of the phenyl moiety then afforded VU0453595 (**15**), a selective M_1_ PAM that found utility as a rodent tool compound for selective M_1_ potentiation, again without M_1_ agonism.^34^ Finally, extensive chemical optimization of the southern benzyl tail region led to the discovery of VU319 (**16**) (Figure 2).

### Discovery and Process Chemistry

**Scheme 1:**
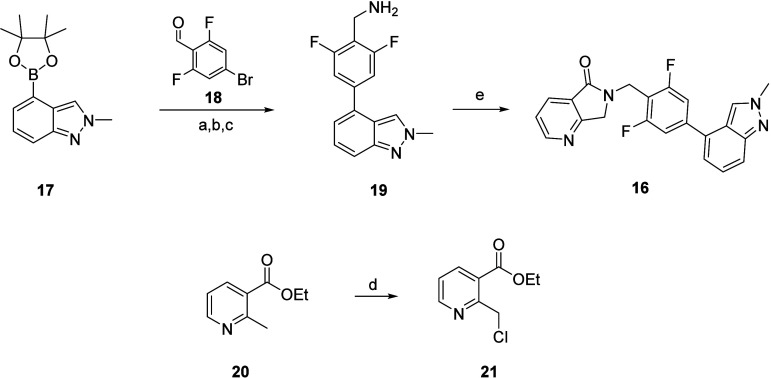
Discovery Chemistry Route for the Synthesis of VU319 (**16**) Reagents and conditions: (a) PdCl2(dppf)·DCM, Cs2CO3, THF:H2O, 40 °C, 12 h; (b) NH2OH·HCl, CH3COONa, EtOH, 2.5 h; (c) Zn, acetic acid, 2.5 h, 79% over three steps; (d) TCCA, DCM, rt, 24 h, 100%; (e) Hünig’s base, CH3CN, 80 °C, 12 h, 40%.

**Scheme 2:**
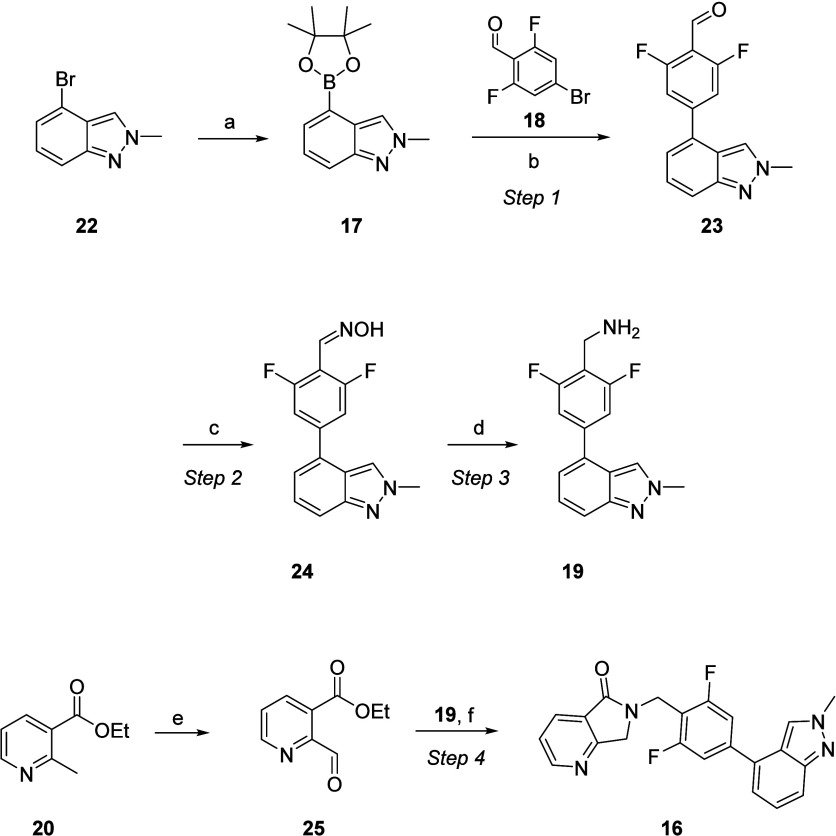
CMC Process Route for the Synthesis of VU319 (**16**) on 3.3 kg Scale Reagents and conditions: (a) Bis(pinacolato)diboron, PdCl_2_(dppf)·DCM, 1,4-dioxane, KOAc, 85–90 °C, 10 h, 66%; (b) PdCl_2_(dppf)·DCM, Cs_2_CO_3_THF:H_2_O, 55–60 °C, 3 h, 95%; (c) NH_2_OH·HCl, CH_3_COONa, EtOH, 5 h, 95%; (d) Zn, acetic acid,2.5 h, 66%; (e) SeO_2_, 1,4-dioxane, 135–140 °C, 2 h, 51%; (f) STAB, ZnCl_2_, THF, 50–55 °C, 6 h, 73%.

For the synthesis of VU319 we developed two synthetic routes: (1) the optimized medicinal chemistry route and (2) the process route employed in the CMC tox lot and GMP supply campaigns.^35^ The optimized discovery chemistry route to VU319 was a convergent route employing commercially available starting materials (Scheme 1). Staring with indazole boronic ester **17**, a Suzuki coupling with bromobenzaldehyde **18** proceeded smoothly, followed by conversion to the oxime and Zn-mediated reduction to key benzylamine **19** in 79% overall yield for the three-step sequence. In parallel, commercial nicotinic ester **20** was chlorinated with trichloroisocyanuric acid (TCCA) to deliver **20** in quantitative yield. Finally, amine **19** was reacted with benzyl chloride **21** in the presence of Hünig’s base at 80 °C leading to lactam formation and the production of VU319 in 40% yield. Overall, the convergent discovery synthesis of VU319 proceeded in five steps with an overall yield of 31.6%.^35^ The CMC process route for a 1.3 kg tox lot and GMP manufacture of a 3.3 kg lot employed a similar route, but key reactions were changed to avoid potential genotoxic intermediates (Scheme 2). Boronic acid **17**, while cost-effective in the discovery chemistry campaign, proved too costly, and difficult to obtain in the necessary quantities, to employ directly as a starting material in the CMC campaign. Thus, commercial bromoindazole **22** was designated starting material 1 (SM 1) and converted into **17** under standard conditions in excellent yield. The first GMP step proceeded with the Suzuki coupling of **17** with **18** (starting material 2, SM 2) under the discovery chemistry conditions to deliver aldehyde **23**. The second GMP step was conversion to the oxime to provide intermediate 3 (**24**), followed by step 4, the Zn-mediated reduction to the benzyl amine **19** (step 3). The benzyl chloride intermediate **21** in the discovery route was flagged as a potential genotoxic intermediate, so the team revised the final coupling steps. Ultimately, commercial nicotinic ester **20** (starting material 3, SM 3) was oxidized with selenium dioxide to the corresponding aldehyde **25**. A reductive amination protocol with **19** and **25** employing STAB (step 4) produced VU319 on scales up to 3.3 kg under cGMP as a white solid. Crude VU319 was recrystallized from ethanol at 60–65 °C. Product was acquired over only four GMP steps in 99.7% purity and with individual impurities ≤0.10% and an overall yield of 50.5% (3.3 kg).^35^ VU319, 6-(2,6-difluoro-4-(2-methyl-2*H*-indazol-4-yl)benzyl)-6,7-dihydro-5*H*-pyrrolo[3,4-*b*]pyridin-5-one, had a clean, sharp melt at 208 °C, a molecular weight of 390.4, an experimental logD_7.4_ of 3.28 and a p*K*_a_ of 1.4.

### Molecular Pharmacology

After two decades of research with M_1_ allosteric agonists,^36−38^ and M_1_ PAMs with minimal to substantial levels of agonism,^15,17,20−22,26,31−34^ we felt that a translatable candidate had to avoid overstimulation of the M_1_ receptor, in order to prevent cholinergic side effects and diminished efficacy at high doses and/or with chronic administration. Our team determined that we needed to develop an M_1_ PAM with minimal agonism in both our stably transfected, TET-inducible mAChR cell lines as well as in native tissues (induction of long-term depression, LTD).^15,17,20−22,26,31−34^ Moreover, the vast majority of M_1_ PAMs that were advanced into clinical testing were very potent, but with low *K*_p_*s*/*K*_p,uu_*s*, typically less than 0.1, leading to overstimulation of the M_1_ receptor (particularly peripheral M_1_ receptors), Racine scale 4/5 seizures in mice, robust induction of LTD in native tissues, cholinergic side effects and diminished pro-cognitive efficacy.^17,19^ Moreover, when we began investigating allosteric modulation of GPCRs, we arbitrarily selected an EC_20_ concentration of acetylcholine as the subthreshold level of endogenous agonist for which to assess PAM activity (i.e., we desired a large PAM window).^31^ In vivo, cholinergic tone/ACh levels vary across brain regions, in different disease states and numerous other caveats; therefore, it is possible that the in vivo potency of our M_1_ PAMs could have been underestimated by the cell-based functional assays.^17^ As a result, our strategy was to translate a moderately potent M_1_ PAM in our cell-based assay, with minimal M_1_ agonism, good CNS penetration and overall drug-like properties to potentiate whatever ACh tone is present in vivo. VU319 delivered on this unique and desired profile.

**Figure 3:**
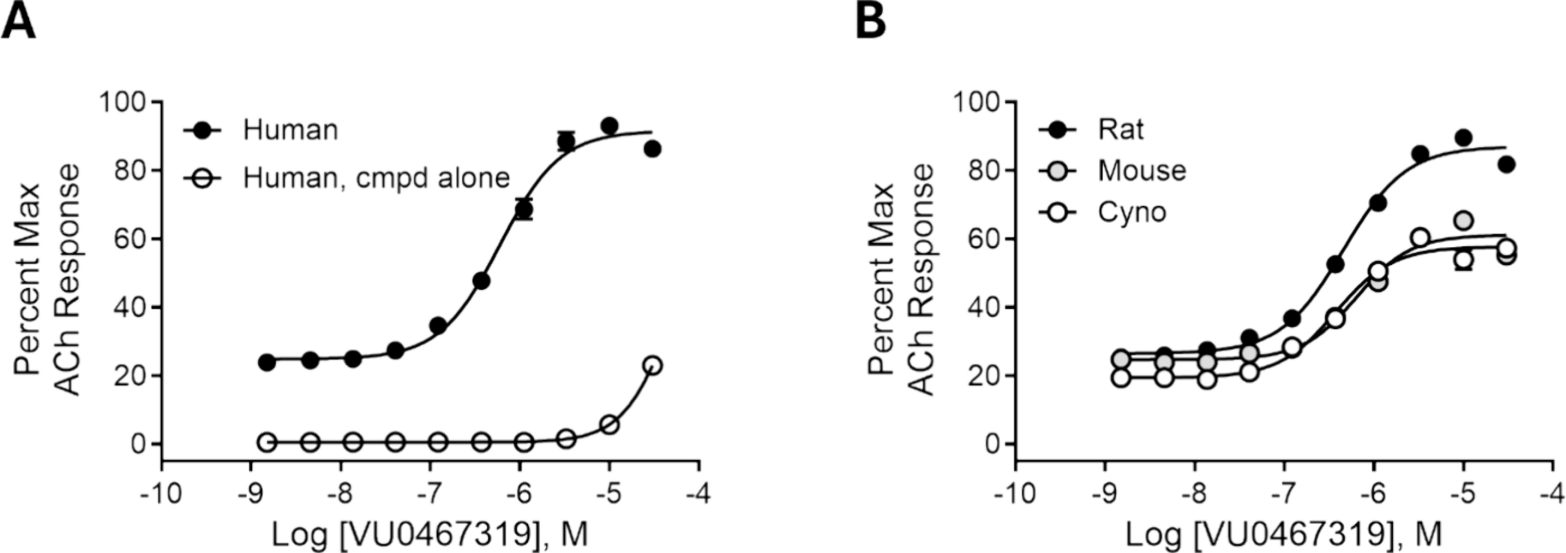
(A) VU319 is a PAM of the human M_1_ muscarinic receptor with minimal M_1_ agonism. Data represent one experiment performed in triplicate. (B) VU319 is a PAM of the rat, mouse, and cynomolgus monkey M_1_ muscarinic receptors. Representative data from one experiment for each species performed in triplicate.

**Figure 4:**
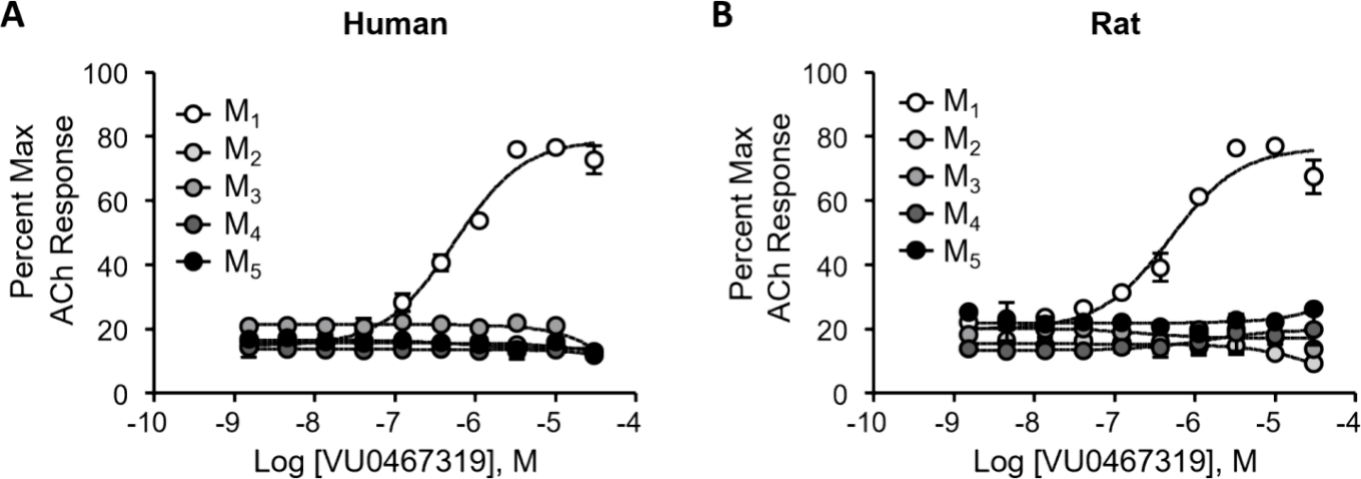
VU319 is highly selective as an M_1_mAChR PAM at human (A) and rat (B) M_1_–M_5_ receptors. Increasing concentrations of VU319 were applied approximately 2 min prior to an appropriate concentration of acetylcholine (ACh) designed to elicit an EC_20_ response. Calcium mobilization responses were measured and normalized to a maximal ACh response elicited after activation of each receptor.

Figure 3A highlights the human M_1_ PAM concentration–response-curve (CRC) in the presence of an ∼ EC_20_ of ACh (M_1_ PAM EC_50_ = 492 nM ± 2.92 nM, 71.3 ± 9.9% ACh Max, *n* = 94) as well as the effect on M_1_ receptor activation in the absence of ACh (ie., the M_1_ agonist CRC).^35^ At a concentration of 30 μM, there is only slight activation, highlighting that VU319 is a PAM with minimal M_1_ agonism (M_1_ agonism >30 μM). As allosteric sites may not be conserved across species, we evaluated the ability of VU319 to potentiate mouse, rat, and cynomolgus monkey (cyno) M_1_ (Figure 3B) to enable translation. Here, VU319 potentiated M_1_ in all three species: rat (M_1_ EC_50_ = 398 ± 195 nM, 81.3 ± 11.3% ACh Max, *n* = 13), mouse (M_1_ EC_50_ = 728 ± 184 nM, 55.9 ± 5.6% ACh Max, *n* = 2 in triplicate) and cyno (M_1_ EC_50_ = 374 nM, 57.8% ACh max, n = 1 individual experiment performed in duplicate). Moreover, PAM **16** was selective (EC_50_ > 30 μM) versus M_2–5_ for both human (Figure 4A) and rat (Figure 4B).^35^

**Figure 5:**
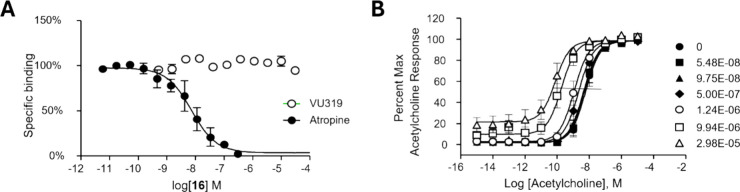
(A) In contrast to atropine, VU319 does not displace the binding of the orthosteric radioligand, [^3^H] NMS. (B) Fold shift of the ACh concentration–response curve in a calcium assay in cells expressing the rat M_1_ receptor and exposed to increasing concentrations of VU319 prior to a full range of ACh concentrations. Data represent two experiments performed in duplicate or triplicate.

In a competitive radioligand binding assay, VU319 did not displace orthosteric [^3^H] *N*-methylscopolamine (NMS) binding at the muscarinic receptors (up to 30 μM), indicating an allosteric mechanism of action (Figure 5A).^35^ In contrast, the orthosteric antagonist atropine displaced [^3^H]-NMS binding with a *K*_i_ of 1.8 nM, in accord with literature values. As shown in Figure 5B, VU319 shifted the ACh concentration–response curve to the left in a calcium mobilization assay in cells expressing the rat M_1_ receptor, with a maximum leftward shift of 96-fold at 30 μM (and 38-fold at 10 μM). PAMs act via increasing the affinity (α cooperativity factor), efficacy (β cooperativity factor), or both of an endogenous orthosteric agonist such as ACh. Here, increasing concentrations of **16** progressively shifted the ACh displacement curve to the left in CHO cells expressing the rat M_1_ receptor. This resulted in a calculated α value of 59, (see Supporting Information) suggesting that part of the mechanism of receptor potentiation induced by VU319 is via an increase in ACh affinity at M_1_. Moreover, and in contrast to the results observed at M_1_, VU319 was ineffective in shifting ACh affinity at rat M_2_, M_3_, M_4_ or M_5_ receptors. An early exploration of signal bias discovered that VU319 also potentiated the recruitment of β-arrestin2 to the human M_1_ receptor in a concentration dependent manner (EC_50_ = 890 nM, 72% max).^35^ Finally, we assessed probe dependence of VU319 and its ability to potentiate other orthosteric agonists. In addition to ACh, VU319 also potentiated the response of oxotremorine-M (M_1_ EC_50_ = 400 nM, 59% ACh Max, M_2–5_ EC_50_ > 30 μM), but was ineffective at potentiating xanomeline, clozapine or *N*-desmethylclozapine (NDMC).^35^

### Drug Metabolism and Pharmacokinetics

VU319 displayed a very attractive in vitro and in vivo DMPK profile, and for the time (ca. 2016–2017) demonstrated improved CNS penetration relative to other M_1_ PAMs reported.^35^ In rodents, VU319 displayed good unbound fraction in both plasma (*f*_u_ (rat, mouse) = 0.028, 0.028) as well as brain homogenate binding (BHB *f*_u_ (rat, mouse) = 0.040, 0.048). Similarly, human (plasma *f*_u_ = 0.034), dog (plasma *f*_u_ = 0.076) and cyno (plasma *f*_u_ = 0.059) all displayed acceptable plasma protein binding. Moreover, VU319 appeared stable in vitro, with low predicted hepatic clearance values across species (CL_hep_ (human, rat, dog cyno): 1.3, 5.7, 5.5, 5.6 mL/min/kg) and an acceptable CYP_450_ inhibition profile (3A4, 2B6, 2D6: IC_50_ > 30 μM; 1A2: IC_50_ = 14 μM; 2C9: IC_50_ = 5.1 μM). Follow-up studies showed no significant CYP_450_ (3A4, 2B6 and 1A2) induction liability, and CYP_450_ phenotyping indicated CYP3A4 solely contributed to the metabolism of VU319. In terms of predicted CNS penetration in man, VU319 was not a human P-gp substrate (MDCK-MDR1 ER = 1.6) or MDCK-BCRP substrate (ER = 1.8) with high permeability (*P*_app_ = 31 × 10^–6^ cm/s). In the FDA transporter panel, no issues arose, including BSEP (IC_50_ > 30 μM). Mini-AMES was negative in two strains (TA98 and TA100 with and without S9), and GSH trapping studies in microsomes across species (h, r, c, d) found only a trace (<0.1%) of adducts.^35^ These positive data paved the way to initiate in vivo PK studies.

In vivo, VU319 displayed significantly better brain exposure in rodents than other PAMs of the time (plasma:brain partitioning *K*_p_*s* < 0.1; *K*_p,uu_*s* ≪0.1). In mouse, VU319 provided a *K*_p_ of 0.77 and a *K*_p,uu_ of 1.3, while in rat, VU319 displayed a *K*_p_ of 0.64 and a *K*_p,uu_ of 0.91.^35^ M_1_ PAMs at the time were very potent, but had low *K*_p_ values, leading to relatively low CNS exposures and cholinergic AEs.^17^ The goal was to provide higher CNS exposure, but with moderate M_1_ PAM potency to effectively potentiate varying ACh tone across brain regions.

Multispecies IV/PO PK studies further supported the low in vitro predicted hepatic clearance (Table 1). Across mouse, rat, dog and cyno, there was a robust in vitro:in vivo correlation (IVIVC) with VU319 showing low clearance (Cl_p_ (m, r, d, c): 25.4, 3.0, 4.0, and 3.3 mL/min/kg), low to moderate volumes (*V*_ss_ 0.67–2.2 L/kg) and attractive half-lives (*t*_1/2_ (m, r, d, c): 4.1, 3.0. 7.5 and 4.3 h). Despite modest solubility (FaSSIF, 7 μM and SGF, 36 μM), VU319 was readily absorbed across species affording early *T*_max_ (1–2 h) and with excellent oral bioavailability (%*F* (m, r, d, c): 80, 93, 100, and 59).^35^ Thus, VU319 possessed an attractive profile for continued derisking and development toward a clinical candidate.

**Table 1:** IV/PO Pharmacokinetic Parameters of **16**

parameter	mouse	rat (SD)	dog (beagle)	NHP (cyno)
dose (mg/kg) iv/po	1/3	1/3	1/3	1/3
CL_p_ (mL/min/kg)	25.4	3.0	4.0	3.3
*V*_ss_ (L/kg)	2.2	0.67	2.1	0.90
elimination *t*_1/2_ (h)	4.1	3.0	7.5	4.3
*F* (%) po	80	93	100	59
*C*_max_ (μM)	3.1	4.5	3.3	3.7
*T*_max_ (h)	1	1	2	2
AUC (μM*h)	39	43	47	23
*K*_p_	0.77	0.64		
*K*_p,uu_	1.3	0.91		

### Derisking Cholinergic AEs

**Figure 6:**
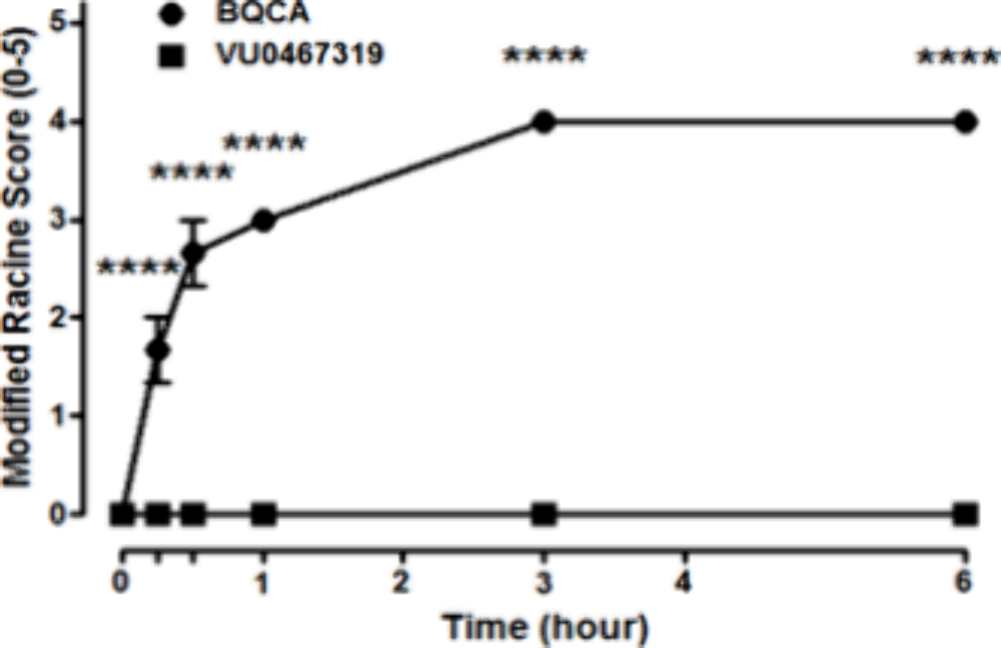
Racine Score test in mice. Pretreatment with M_1_ PAMs (100 mg/kg, i.p., 10 mL/kg, 180 min) BQCA (**2**) resulted in robust behavioral convulsions at 30 min post administration, while VU319 did not cause any observed adverse effects out to 6 h post administration. *N* = 3/group of male C57Bl/6 mice. ANOVA *p* < 0.0001; ****p < 0.0001 as compared to vehicle control.

**Figure 7:**
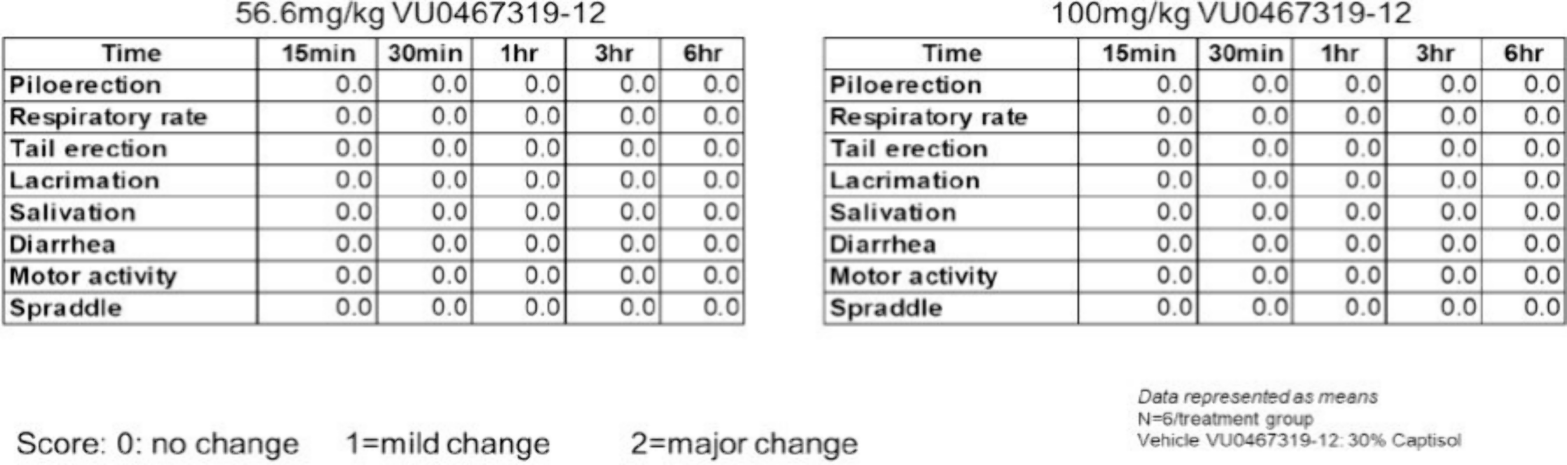
Effects of VU319 on the modified Irwin Neurological Test battery in mice. At doses of either 56.6 mg/kg or 100 mg/kg IP, and over a 6 h time course, no adverse cholinergic events (SLUDGE) were noted.

In our optimization work-flow, we incorporate a high-throughput phenotypic seizure liability assay (100 mg/kg intraperitoneal (i.p.)), as mice are very sensitive to cholinergic mechanisms and they readily display Racine scale seizures when the M_1_ receptor is over stimulated.^26,39,40^ This is a high-bar assay, as this sets a very conservative exposure for initiation of cholinergic AEs. Here (Figure 6), a high dose (100 mg/kg intraperitoneal (IP)) of BQCA (**2**), a potent M_1_ PAM significant M_1_ agonism,^13−15^ rapidly initiated Racine scale 3/4 seizures 30 min post administration. In contrast, VU319 did not induce seizure liability up to 6 h post administration, consistent with other M_1_ PAMs, with minimal M_1_ agonism. A satellite IP PK study (100 mg/kg IP in C57Bl6 mice, 30% captisol) demonstrated that total brain exposures were 85.4 μM (117-fold above the mouse M_1_ EC_50_).^35^ As discussed in the past, pharmacodynamic effects for dozens of M_1_ PAMs correlates more closely with total brain than with free brain concentrations, as the arbitrarily selected EC_20_ value for the PAM EC_50_ likely underestimates endogenous ACh tone. To assess peripheral cholinergic toxicity, we performed a modified Irwin neurological battery to score autonomic or somatosensory side effects (Figure 7). At a dose of 56.6 mg/kg IP, BQCA (**2**) produced significant SLUDGE effects in mice. In contrast, VU319 at doses of either 56.6 mg/kg IP (60.4 μM, 83-fold above the mouse M_1_ PAM EC_50_) or at 100 mg/kg IP (85 μM, ∼ 117-fold above the mouse M_1_ EC_50_), had no SLUDGE noted.

In a Eurofins Lead Profiling radioligand panel of 68 GPCRs, ion channels and transporters tested at 10 μM, VU319 only displaced radioligand binding at two (α_2a_, *K*_i_ = 2.7 μM and imidazoline I_2_ central, *K*_i_ = 2.8 μM) of the 68 targets. In follow-up functional assays, VU319 was inactive at both targets.^35^ Moreover, VU319 did not displace hERG radioligands, but in a functional EP assay, VU319 displayed an IC_50_ of 12 μM (a value we could go forward with).

A hallmark of potent M_1_ PAMs with intrinsic M_1_ agonism is M_1_ receptor overstimulation and induction of Long-Term Depression (LTD) in layer V medial prefrontal cortex (mPFC).^26^ Therefore, we wanted to explore if VU319 would be devoid of LTD-induction. In electrophysiological studies in native mouse tissues, VU319 did not induce any significant change in field excitatory post synaptic potentials (fEPSPs) recorded from layer V and evoked by electrical stimulation in layer II/III at either 10 μM (∼14× above the functional mouse EC_50_), or 30 μM concentrations (∼42× above the functional mouse EC_50_). Thus, VU319 maintains activity dependence of PFC function, is devoid of seizure liability and does not show induce SLUDGE, in mice up to 100 mg/kg, indicating the absence of cholinergic toxicity in the most sensitive species to observe these adverse effects. Moreover, the pharmacological profile of VU319 clearly differentiates from the M_1_ PAMs with high intrinsic M_1_ agonism that dominated the field at the time (**2**-**5**, 2009–2016).^13−26^

### In Vivo Behavior

**Figure 8:**
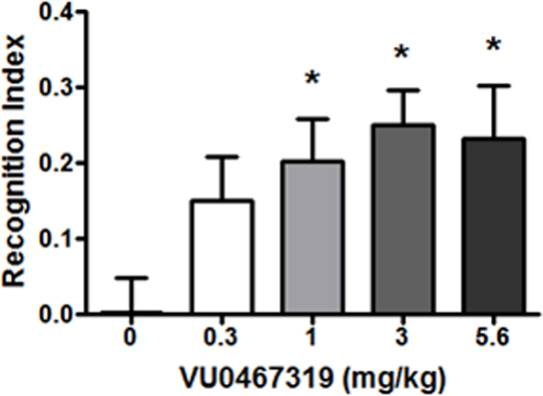
Effects of VU319 on Novel Object Recognition in rats. VU319 dose-dependently enhanced recognition memory in rats. Pretreatment with 0.3, 1, 3, and 5.6 mg/kg VU319 (p.o, 0.5% natrosol/0.015% Tween 80 in water, 30 min) prior to exposure to identical objects significantly enhanced recognition memory assessed 24 h later. *N* = 10–18/group of male Sprague–Dawley rats. ANOVA *p* = 0.0053, *p < 0.05.

VU319 possessed a unique profile of modest M_1_ PAM potency (with minimal M_1_ agonism) while displaying CNS penetration 6–10-fold better than prior M_1_ PAMs **2**-**5**.^13−26^ While the profile of VU319 avoided cholinergic toxicity, would this profile translate to efficacy in preclinical cognition models? Historically, novel object recognition (NOR) has been our first-tier cognition assay to assess M_1_ PAM efficacy.^26^ In the study (Figure 8), pretreatment of VU319 (0.3 to 5.6 mg/kg PO) dose-dependently increased the recognition index during the NOR test in normal Sprague–Dawley rats (*p* = 0.0053, *p < 0.05) relative to the vehicle-treated control group (*N* = 10–18). The minimum effective dose (MED) was 1 mg/kg PO (1 μM total brain, ∼ 2.5× the rat M_1_ PAM EC_50_, *K*_p_ = 0.82), with a trend at 0.3 mg/kg (400 nM total brain, ∼ 1× the rat EC_50_).^35^ Maximum recognition index was achieved between 3 and 5.6 mg/kg (3.2 to 7.2 μM total brain, ∼ 7–16× the rat EC_50_). Thus, an M_1_ PAM of modest potency (rat EC_50_ = 398 nM, 81% ACh Max) with high CNS penetration proved to be efficacious at low doses.

There are multiple potential disease states that could potentially benefit from treatment with an M_1_ PAM. Previously we demonstrated the ability of PAM **7** to reverse cognitive deficits induced by the atypical antipsychotic risperidone,^26^ which is associated with cognitive symptom clusters in schizophrenia. Recent work from the Tobin lab suggests a key disease-modifying role in prion diseases,^25^ while the Niswender lab has shown efficacy in Rett syndrome.^41^ Beyond these disorders, multiple forms of dementia (mild cognitive impairment, vascular dementia, Lewey body dementia and Alzheimer’s disease (AD)) are potentially attractive therapeutic indications for an M_1_ PAM.^1−6^

**Figure 9:**
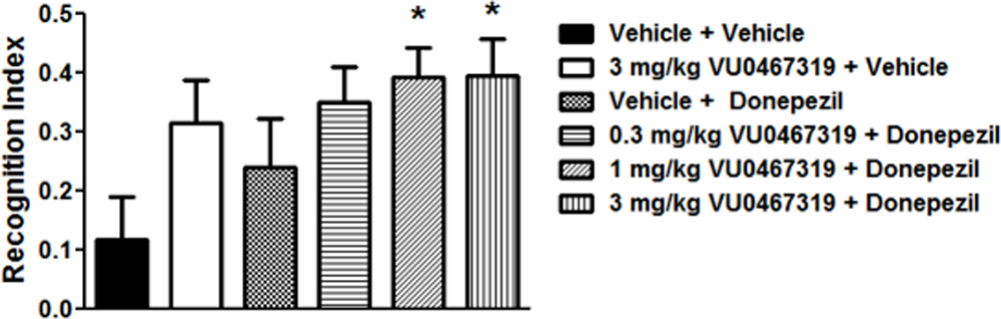
VU319 (PO), in combination with an inactive dose of donepezil (0.3 mg/kg IP) produced enhanced recognition memory in the NOR task in rats. Dose-dependent enhancement of the recognition index during the NOR task (*p* = 0.0449, one-way ANOVA with a dunnett post hoc test * *p* < 0.05) (*N* = 11–12).

Since acetylcholine esterase inhibitors (AChEIs) represent one of the only approved classes of drugs for the treatment of cognitive impairments observed in AD patients,^42^ it was important to also determine the potential additive effects of our M_1_ PAM when given in combination with an AChEI on cognitive functions. We assessed the in vivo efficacy of VU319 in combination with donepezil in the NOR task, a preclinical model of memory function in rats following a single oral administration. Male Sprague–Dawley rats were treated with VU319 formulated as a microsuspension in 20% (w/v) HPBCD in sterile water at concentrations of 0.03, 0.1, or 0.3 mg/mL and administered as a single 0.3, 1, or 3 mg/kg dose (10 mL/kg) by oral gavage. Rats were administered vehicle or VU319 60 min before a vehicle or 0.3 mg/kg intraperitoneal administration of donepezil (1 mL/kg). Data are expressed as mean ± SEM and were analyzed using a one-way ANOVA; if significant (*p* < 0.05), all dose groups were compared with the vehicle-treated group using a Dunnett’s post hoc test. As shown in Figure 9, the dose of 0.3 mg/kg donepezil alone did not significantly increase recognition memory relative to vehicle-treated controls; however, when given in combination with increasing doses of VU319, there was a dose-dependent enhancement of the recognition index during the NOR task (*p* = 0.0449, * *p* < 0.05) (*N* = 11–12). To ensure this synergy was not due to a drug–drug interaction (DDI), we preformed parallel PK studies to assess both agents as DDI perpetrators and victims. Fortunately, there was no DDI for either drug. Collectively, these data suggest that selective activation of M_1_ by VU319 may enhance the efficacy of AChEIs when given in combination in clinical populations.

Beyond these assays, VU319 dose-dependently reversed scopolamine-induced deficits in the acquisition of contextual fear conditioning (CFC), dose-dependently reversed scopolamine-induced deficits in the eight arm radial arm maze (RAM) task, dose-dependently reversed risperidone-induced deficits in CFC, increased active wake 1–2 h post dosing in EEG without impact on any other sleep parameters (consistent with cognitive promoting effects without disrupting sleep quality), but was inactive, as anticipated in reversing amphetamine-induced hyperlocomotion.^35^ Finally, we derisked other potential liabilities in vivo. VU319 has no disruptive effects on basal locomotor activity and motor coordination (rotorod) in rats at doses up to 10 mg/kg (10× above the MED in PD models).^35^

**Figure 10:**
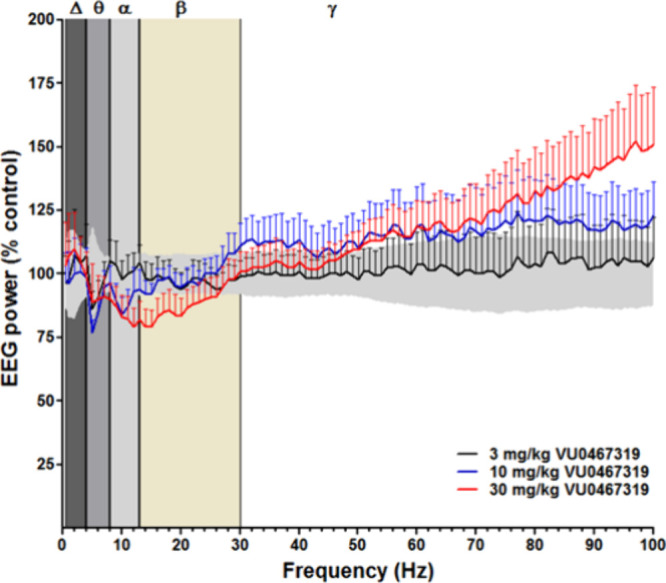
VU319 (PO) produced increases in high gamma (γ) power in rats in a dose-dependent manner. For all dose–response studies a two-way analysis of variance was applied to examine effects of dose and frequency for each of the frequency bands; significance was defined as *p* < 0.05.

As we began to consider biomarker strategy, we knew PET would not work, as M_1_ affinity was low, but cooperativity was very high (leading to the nM functional PAM potency). Moreover, we were never able to discover and develop M_1_ Negative Allosteric Modulators (NAMs) to employ as radioligands and PET tracers competitive with VU319. Thus, the team explored possible functional biomarkers such as FMRI and EEG, and though not quantitative, these could potentially provide qualitative target engagement in Phase I. As shown in Figure 10, VU319 treatment decreased alpha and beta frequencies and increased both the low and high frequency gamma power in the frontal cortex, specifically when the rats were awake (*p* < 0.0001). However, VU319 treatment did not disrupt sleep quality as indicated by no change in delta power during SWS. During wake, pretreatment with PAM VU319 did not alter the power of either delta (*p* = 0.8910) or theta (*p* = 0.3495) frequencies at 1–2 h after dosing.^35^ Taken together, these data reveal that VU319 produces enhanced levels of arousal during wake, consistent with cognitive promoting effects, without disrupting sleep quality.

### Metabolite Identification

**Figure 11:**
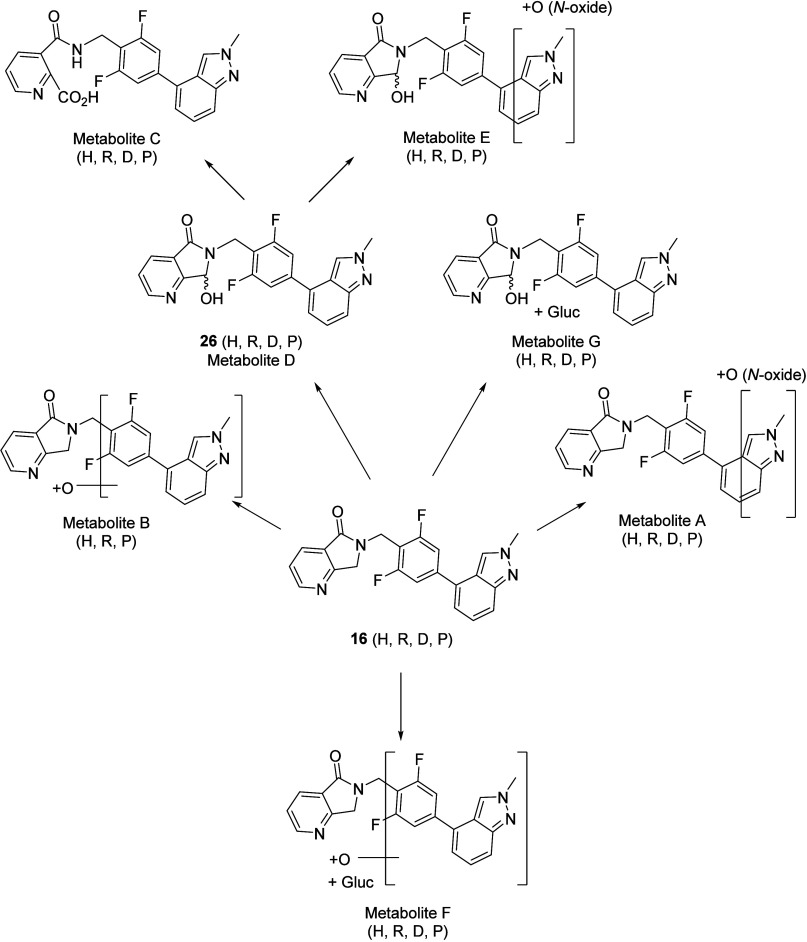
Multispecies hepatocyte metabolite identification of VU319. Metabolite D (VU0481424, **26**) is a major, inactive metabolite.

**Scheme 3:**
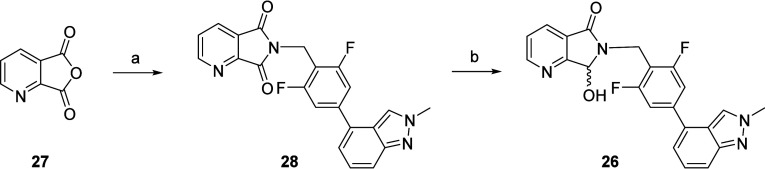
Synthesis of Metabolite D (VU0481424, **26**) Reagents and conditions: (a) **19**, Et_3_N, DMF/MeCN, rt, 1 h, then HATU, 90%; (b) NaBH_4_, DCM/MeOH, 24%.

With enthusiasm for VU319 as a candidate, there remained a few additional sets of data needed to confirm its candidacy. First and foremost, we needed to ensure that we had metabolite coverage across our IND-toxicology safety species and that there were no unique human metabolites. Preliminary biotransformation work in multispecies liver S9 microsomes was later confirmed with VU319 incubations in human (H), rat (R), dog (D) and monkey (P, primate) hepatocytes. A total of seven metabolites were identified in these studies (Figure 11). VU319 was the major component in human (65%), rat (76%) and dog (71%), but lower in monkey (25%) by UV absorbance peak area. In all species, Metabolite D (VU0481424, **26**) was the most abundant metabolite: human (34%), rat (23%), dog (28%) and monkey (74%). The other metabolites were of very low abundance (<0.3%). Importantly, there were unique human metabolites. As VU0481424 (Metabolite D, **26**) was produced in significant quantities, we had to synthesize it (Scheme 3) and assess its pharmacology and DMPK profiles. The synthesis of **26** proved straightforward. Starting from commercial anhydride **27**, treatment with benzyl amine **19** and triethylamine followed by HATU coupling conditions afford the succinimide congener **28** in 90% yield. Several reductants for the chemoselective reduction of **28** to afford **26** were evaluated; ultimately, NaBH_4_, possibly via chelation to the pyridine nitrogen, provided **26** in 24% yield.^35^

Metabolite D (**26**) proved to be an inactive metabolite (human M_1–5_ EC_50_*s* > 10 μM); however, it proved to have a good DMPK profile, despite having an acylated aminal functional group. Like the parent VU319, **26** displayed good unbound fraction in both rat plasma (*f*_u_ = 0.038) as well as brain homogenate binding (BHB *f*_u_ = 0.046). Similarly, human (plasma *f*_u_ = 0.063), dog (plasma *f*_u_ = 0.12) and cyno (plasma *f*_u_ = 0.06) all displayed acceptable plasma protein binding. Moreover, **26** appeared stable in vitro, with low predicted hepatic clearance values across species (CL_hep_ (human, rat, dog cyno): 11.2, 34.5, 11.7, 16 mL/min/kg) and an acceptable CYP_450_ inhibition profile (3A4, 2B6, 2D6: IC_50_ > 30 μM; 1A2: IC_50_ = 0.19 μM; 2C9: IC_50_ = 13 μM), except for 1A2. The metabolite **26** was also brain penetrant, with a rat *K*_p_ of 0.29 and a *K*_p,uu_ of 0.35, and an excellent rat PK profile (Cl_p_ = 7.9 mL/min/kg, *V*_ss_= 1.4 L/kg, *t*_1/2_ = 4 h). Ancillary pharmacology profiling was clean, so there were no pharmacological concerns with the presence of **26**, but we would have to monitor it in the 28-day IND toxicology and in the Phase I SAD clinical trial.

The last remaining data needed were human dose projections, to determine if both development and cost of goods for VU319 was warranted. With the in vitro and in vivo multispecies DMPK data for VU319 in hand, and exposures/effective concentrations of VU319 from the NOR assay, we evaluated human PK parameters and dose projections using six different methods (Table 2). The average of all six methods predicted VU319 to be a low clearance compound in man (CL_p_ < 1.3 mL/min/kg)) with a reasonable volume (*V*_ss_ 0.52–1.2 L/kg) and a long half-life (up to 12 h). Projected human doses ranged from 150 to 440 mg for 24 h QD coverage to 50–100 mg for 24 h BID. A more likely scenario would be 12–16 h coverage, where projected QD doses range from 75 to 195 mg, or BID dose range from 40 to 70 mg. As these are not steady state PK approximations, dose projections could improve based on actual human PK in Phase I. Moreover, if given in combination with donepezil, it theoretically might be possible to use a lower dose of VU319.

**Table 2:** Human PK and Efficacious Dose Projections for VU319

compound (ID)	prediction method	predicted human PK parameter			projected human efficacious dose (mg)^a^					
					24 h coverage^b^		16 h coverage^c^		12 h coverage^d^	
		CL_p_ (mL/min/kg)	*V*_ss_ (L/kg)	*t*_1/2_ (h)	QD (24 h tau)	BID (12 h tau)	QD (24 h tau)	BID (8 h tau)	QD (24 h tau)	BID (6 h tau)
VU0467319	HLM^e^	1.3	0.76	6.8	440	100	195	70	135	65
	HLM R/D/C IVIVE^f^	0.79	0.76	11	150	50	90	40	75	40
	SSS rat^g^	0.93	0.81	10	195	60	115	50	85	45
	SSS dog^g^	1.1	0.94	10	235	70	135	60	105	55
	SSS cyno^g^	0.98	0.52	6.1	385	80	160	55	115	50
	simple R/D/C allometry^h^	1.1	1.2	12	190	65	125	60	100	55
	all (range)	0.79–1.1	0.52–1.2	6.1–12	150–440	50–100	90–195	40–70	75–135	40–65

a Mean K_a_ from rat and cyno oral PK studies (∼0.7/h, MAT approach) and oral F from cyno (0.59, lowest) used for all prediction methods (ref: Chiou et al. 1998; 2000 Pharm. Res. 15:11 and 17:2).

b Doses projected to provide 24 h daily coverage of the targeted *C*_min,p_ based on the *C*_max,p_ from rat NOR model MED [1 mg/kg PO] corrected for species differences in fu_plasma_, and in vitro potency.

c Doses projected to provide 16 h daily coverage of the targeted *C*_min,p_, based on the *C*_max,p_, from rat NOR model MED [1 mg/kg PO] corrected for species differences in fu_plasma_ and in vitro potency.

d Doses projected to provide 12 h daily coverage of the targeted *C*_min,p_, based on the *C*_max,p_ from rat NOR model MED [1 mg/kg PO] corrected for species differences in fu_plasma_ and in vitro potency.

e Direct scaling of CL from human liver microsomes using the well-stirred model with fu_plasma_ and calculated fu_mics_ predicted human *V*_ss_ is mean from rat/dog/cyno SSS predictions.

f Scaling of CL_int_ from human liver microsomes according to the same method as described for “method a” but with correction for the mean fold-difference observed between in vitro and calculated in vivo unbound CL_int_ in rat, dog, and cyno (approximately ∼0.6x); predicted human *V*_ss_, is mean from rat/dog/cyno SSS predictions.

g Single-species allometric scaling of rat, dog, or cyno CL_p_, (exp. 0.75) and *V*_ss_ (exp. = 1), respectively, corrected for species differences in fu_plasma_.

h Multispecies allometric scaling of CL_p_ and *V*_ss_ (using empirically determined exponents) corrected for species differences in fu_plasma_.

### Investigational New Drug (IND)-Enabling Studies

Based on the overall profile of VU319, and with a generous grant from the William K. Warren Foundation to support IND-enabling studies, we contracted with Davos Pharma for the CMC (Scheme 2), IND-enabling toxicology and regulatory services to draft the Investigational New Drug Application and take VU319 to an open IND. VU319 was negative in the standard IND battery of in vitro genotoxicity assays, no treatment-related findings were noted in respiratory rat, and no effect on any cardiac parameter was observed in CV dog. Dose-range finders (DRFs) and maximum tolerated dose (MTD) studies were conducted in male and female cohorts of rats, dogs and nonhuman primates, achieving exposures 10- to 20-fold over the rat NOR MED exposure (and >25-fold over the M_1_ PAM EC_50_) without any sign of SLUDGE or toxicity findings in the non-GLP histopathology analysis. GLP 28-day toxicology was performed in rat and dog, achieving exposures >20-fold the rat NOR MED without SLUDGE or observable AEs. The only clinical sign noted was mild weight loss, from which the animals fully recovered.

These data were compiled and submitted to the FDA as an Investigational New Drug (IND) Application on October 3, 2016, and the IND was opened on November 2, 2016. With the IND data package, and in collaboration with Dr. Paul Newhouse, the Director of the Center for Cognitive Medicine at Vanderbilt, a first-in-human Phase I single ascending dose (SAD) study with VU319 was initiated in the Vanderbilt Institute for Clinical and Translational Research (VICTR). NCT03220295 was initiated on July 18, 2018 (almost a decade after we discovered HTS hit **11**).^29^ Subsequently, safety and pharmacokinetic data from that trial has been reported.^27,28^ In human volunteers, VU319 was well tolerated, with no cholinergic side effects (SLUDGE) noted, at doses showing signs of cognitive improvement and functional target engagement. A full account of the Phase I SAD VU319 is in preparation and will be published in due course.

## Conclusions

In summary, a lead optimization campaign starting from a chemically reactive, nonselective M_1,3,5_ PAM, with minimal M_1_ agonism, was converted into VU319, a highly selective (>30 μM versus M_2–5_) and highly brain penetrant (rat *K*_p_ of 0.64, *K*_p,uu_ of 0.91) M_1_ PAM (EC_50_ = 492 nM, 71% ACh Max), while retaining the desired minimal M_1_ agonism. Relative to prior M_1_ PAMs that had poor CNS penetration (*K*_p_*s* < 0.1) and cholinergic side effects, we opted for an M_1_ PAM profile of modest potency, minimal agonism and high CNS penetration. VU319 was efficacious in multiple preclinical cognition models (MEDs of 1 mg/kg PO) and demonstrated no cholinergic AEs in mice (the most sensitive species) or rats, suggesting our strategy was viable. VU319 successfully navigated IND-enabling studies with no alerting genotoxicity, safety pharmacology or repeat-dose oral toxicity liabilities identified at the achieved nonclinical VU-319 doses/exposures, and was devoid of cholinergic AEs in rats, dogs and NHPs. To assess tolerability and pharmacokinetics in humans, VU319 entered a Phase I SAD trial in 2018 (NCT03220295), and was found to be well tolerated with no evidence of cholinergic toxicity (SLUDGE) at doses/exposures where signs of human pro-cognitive benefit were demonstrated. The discovery of the VU319 marks a very important achievement, demonstrating the potential for selective M_1_ PAMs to engage central M_1_ receptors in humans without causing cholinergic side effects.

## Supporting Information Available

Additional experimental details, methods for the synthesis and characterization of all compounds, in vitro and in vivo DMPK protocols, and supplemental figures (PDF)

## Abbreviations

PAM: positive allosteric modulator
PBL: plasma/brain level
DMPK: drug metabolism and pharmacokinetics
AE: adverse event
M_1_: muscarinic acetylcholine receptor subtype 1
MED: minimum effective dose
NOR: novel object recognition
IND: investigational new drug
FDA: federal drug administration
