# Supplementary material for: Discovery of VU0467319: an M1 Positive Allosteric Modulator Candidate That Advanced into Clinical Trials
Source: ACS Chem Neurosci. 2024 Dec 11;16(1):95–107. doi: 10.1021/acschemneuro.4c00769 (PMC11697341; doi:10.1021/acschemneuro.4c00769)
Supplement: Supplementary file 1 — cn4c00769_si_001.pdf [file cn4c00769_si_001.pdf]

Discovery of VU0467319: an M_1 Positive Allosteric Modulator Candidate that Advanced into Clinical Trials

Michael S. Poslunsey,[1,2] Michael R. Wood,[1,2] Changho Han,[1,2] Shaun R. Stauffer,[1,2] Joseph D. Panarese,[1,2] Bruce J. Melancon,[1,2] Julie L. Engers,[1,2] Jonathan W. Dickerson,[1,2] Weimin Peng,[1,2] Meredith J. Noetzel,[1,2] Hyekyung P. Cho,[1,2] Alice L. Rodriguez,[1,2] Corey R. Hopkins,[1,2] Ryan Morrison,[1,2] Rachel D. Crouch,[1,2] Thomas M. Bridges,[12] Anna L. Blobaum,[1,2] Olivier Boutaud,[1,2] J. Scott Daniels,[1,2] Michael J. Kates,[7] Arlindo Castelhano,[7] Jerri M. Rook,[1,2] Colleen M. Niswender,[1,2,4,5,6] Carrie K. Jones,[1,2] P. Jeffrey Conn,[1,2,4] and Craig W. Lindsley*[1,2,3,4]

Affiliation:
[1]Warren Center for Neuroscience Drug Discovery, Vanderbilt University, Nashville, TN 37232, USA
[2]Department of Pharmacology, Vanderbilt University School of Medicine, Nashville, TN 37232, USA
[3]Department of Chemistry, Vanderbilt University, Nashville TN 37232, USA
[4]Vanderbilt Kennedy Center, Vanderbilt University Medical Center, Nashville, TN 37232, USA
[5]Vanderbilt Brain Institute, Vanderbilt University, Nashville, TN 37232, USA
[6]Vanderbilt Institute of Chemical Biology, Vanderbilt University, Nashville, TN 37232, USA
[7]Davos Pharma, Upper Saddle River, NJ 07458, USA

*To whom correspondence should be addressed at craig.lindsley@vanderbilt.edu

TABLE OF CONTENTS

Eurofins Lead Profiling Screen....................S2
Procedures for Biological Experiments....................S4
General Methods....................S14
Synthetic Procedures and spectra....................S16
Supplemental Figures....................S26
References....................S36

Table S1. Eurofins Lead Profiling Screen Data

This is a radioligand binding panel of 67 targets including GPCRs, ion channels, transporters and nuclear hormones. Biochemical assay results are presented as the percent inhibition of specific binding at a 10 μM concentration of **VU0467319** (Compound **16**).

**Target/Protein**	**Species**	**% Inhibition at 10 μM**
Adenosine A_1	Human	26
Adenosine A_{2A}	Human	7
Adenosine A_3	Human	7
Adrenergic α_{1A}	Human	-3
Adrenergic α_{1B}	Human	7
Adrenergic α_{1D}	Human	16
Adrenergic α_{2A}	Human	67
Adrenergic β_1	Human	0
Adrenergic β_2	Human	-9
Androgen (Testosterone)	Human	7
Bradykinin B_1	Human	0
Bradykinin B_2	Human	8
Calcium Channel L-Type, Benzothiazepine	Rat	12
Calcium Channel L-Type, Dihydropyridine	Rat	12
Calcium Channel N-Type	Rat	1
Cannabinoid CB_1	Human	12
Dopamine D_1	Human	7
Dopamine D_{2S}	Human	14
Dopamine D_3	Human	0
Dopamine $D_{4.2}$	Human	1
Endothelin ET_A	Human	-5
Endothelin ET_B	Human	4
Epidermal Growth Factor (EGF)	Human	4
Estrogen ERα	Human	15
$GABA_A$, Flunitrazepam, Central	Rat	3
$GABA_A$, Muscimol, Central	Rat	-5
$GABA_{B1A}$	Human	1
Glucocorticoid	Human	8
Glutamate, Kainate	Rat	6
Glutamate, NMDA, Agonism	Rat	19
Glutamate, NMDA, Glycine	Rat	3
Glutamate, NMDA, Phencyclidine	Rat	5
Histamine H_1	Human	10
Histamine H_2	Human	1
Histamine H_3	Human	-4
Imidazoline I_2, Central	Rat	70

Interleukin IL-1 R1	Human	11
Leukotriene, Cysteinyl $CysLT_1$	Human	-3
Melatonin MT_1	Human	10
Muscarinic M_1	Human	19
Muscarinic M_2	Human	0
Muscarinic M_3	Human	15
Neuropeptide Y Y_1	Human	9
Neuropeptide Y Y_2	Human	4
Nicotinic Acetylcholine α3β4	Human	-3
Nicotinic Acetylcholine α1, Bungarotoxin	Human	7
Opiate δ_1 (OP1, DOP)	Human	-8
Opiate κ (OP2, KOP)	Human	6
Opiate μ (OP3, MOP)	Human	-2
Phorbol Ester	Mouse	11
Platelet Activating Factor (PAF)	Human	40
Potassium Channel [K_{ATP}]	Hamster	7
Potassium Channel hERG	Human	5
Prostanoid EP_4	Human	4
Purinergic P2X	Rat	11
Purinergic P2Y, Non-Selective	Rat	14
Rolipram	Rat	19
Serotonin (5-HT_{1A})	Human	-5
Serotonin (5-HT_{2B})	Human	16
Serotonin (5-HT_3)	Human	-3
Sigma σ_1	Human	-8
Sodium Channel, Site 2	Rat	-3
Tachykinin NK_1	Human	10
Thyroid Hormone	Rat	9
Transporter, Dopamine (DAT)	Human	-1
Transporter, GABA	Rat	5
Transporter, Norepinephrine (NET)	Human	30
Transporter, Serotonin (SERT)	Human	4

Summary of Significant Results

Above Primary Tests in Rank Order of Potency

Cat #	Assay Name	Species	Conc.	% Inh.	IC_{50}*	K_i	n_H
203630	Adrenergic α_{2A}	hum	10 μM	61	5.33 μM	2.66 μM	0.77
241000	Imidazoline I_2, Central	rat	10 μM	68	4.14 μM	2.76 μM	0.84

Procedures for Biological Experiments

Calcium mobilization assays:

All functional cell-based assays were performed in stable Chinese Hamster Ovary (CHO) cell lines constitutively expressing human M_5 or human M_1 receptors. For full muscarinic selectivity, CHO cells expressing human M_3, human M_2 plus G_{qi5} or human M_4 plus G_{qi5} were used. Cells were plated at 15,000 cells per 20 µL per well in black 384-well, TC-treated, clear-bottomed plates (Greiner) in Ham's F12 medium supplemented with 10% FBS and 20 mM HEPES. Cells were incubated overnight at 37 °C under 5% CO_2. The following day, the medium was removed and replaced with 1.2 µM Fluo-4 AM (Invitrogen) in assay buffer (Hank's Balanced Salt Solution supplemented with 20 mM HEPES and 2.5 mM Probenecid, pH 7.4) and the cells were incubated for 50 minutes at 37 °C under 5% CO_2. Dye was then removed and replaced with 20 µL of fresh assay buffer. Test compounds at a 10 mM concentration in DMSO were serially diluted in DMSO (either 1:3 or 1:5 dilution) to create a 10-point concentration series. The DMSO solutions were then diluted in assay buffer resulting in compound solutions at 2-times the final assay concentration with the highest assay concentration of 30 µM. The compound plate, cell plate, and plates containing EC_{20} and EC_{80} acetylcholine concentrations were placed in a Hamamatsu FDSS 6000 or 7000 kinetic imaging plate reader equipped to measure Ex_{480}/Em_{540} fluorescence. Data were collected at 1 frame per second. After 2 seconds of collecting baseline fluorescence, 20 µL of the compound solutions were added to the cell plate. This was followed by the addition of an EC_{20} concentration of acetylcholine at 142 seconds. At 267 seconds, an EC_{80} concentration of acetylcholine was added along with a maximally effective acetylcholine concentration in wells not containing a compound to allow data normalization. The fluorescence signal was collected for a total of 300 seconds. Compound concentration response curves (CRCs) were collected in triplicate across three separate plates. Data were imported and analyzed in Dotmatics Informatics software. by normalizing all data in the individual kinetic traces to the initial fluorescence read. The magnitude of each agonist addition was then determined and normalized to the average maximum response. This percent maximum response was plotted against log[compound] and fit to a four parameter logistical equation to determine $\log(IC_{50})$. The IC_{50} determined using the EC_{80} of acetylcholine is the value reported. Compound CRC curves that did not plateau below 10% ACh_{max} were assigned a low potency value of >10 µM.

Drug Metabolism Methods:

In vitro

Plasma protein binding and Brain homogenate binding: Determination of fraction unbound (f_u) in plasma was conducted in vitro via equilibrium dialysis using HTDialysis (HTD) membrane plates. The top half of the plate was filled with 100 uL of Dubelco's Phosphate Buffered Saline, pH 7.4 (DPBS). Compounds were diluted into plasma from each species (5 µM final concentration), which was aliquoted in triplicate to the 'bottom half' of the prepared HTD plate wells. The HTD plate was sealed and incubated for 6 hours at 37 ˚C. Following incubation, each well (both top and bottom halves) were transferred (20 µL) to the corresponding wells of a 96-shallow-well (V-bottom) plate. The daughter plates were then matrix-matched (DPBS side wells received equal volume of plasma, and plasma side wells received equal volume of DPBS), and extraction solution (120 µL; acetonitrile containing 50 nM carbamazepine as IS) was added to all wells of both daughter plates to precipitate protein and extract test article. The plates were then sealed and centrifuged (3500 rcf) for 10 minutes at ambient temperature. Supernatant (60 µL) from each well of the daughter plates was then transferred to the corresponding wells of new daughter plates (96-shallow-well, V bottom) containing water (Milli-Q, 60 µL/well), and the plates were sealed in preparation for LC-MS/MS analysis (see LC-MS/MS analysis method below).

The unbound fraction (f_u) was calculated following the equation below, and mean values for each species were calculated from 3 replicates.

A similar approach was used to determine the degree of brain homogenate binding, which employed the same methodology and procedure with the following modifications: 1) a final compound concentration of 1 µM was used, 2) naïve rat brains were homogenized in DPBS (1:3 composition of brain: DPBS, w/w) using a Mini-Bead Beater™ machine in order to obtain brain homogenate, which was then treated in the same manner as the plasma samples in the previously described plasma protein binding assay. Fraction unbound for both plasma and brain samples was determined using Equation 4.

$$f_u = \frac{Conc_{buffer}}{Conc_{plasma}}$$

Equation 4 Determination of fraction unbound in plasma.

The diluted fraction unbound (f_u2) in brain was calculated in the same manner by using brain homogenate rather than plasma. Undiluted fraction unbound for the brain was calculated using Equation 5

$$f_u = \frac{1/4}{\left\{\left(\frac{1}{fu2}\right) - 1\right\} + 1/4}$$

Equation 5 Determination of fraction unbound in brain. F_u2 represents the diluted fraction unbound.

Intrinsic clearance: Human or rat hepatic microsomes (0.5 mg/mL) and 1 µM test compound were incubated in 100 mM potassium phosphate pH 7.4 buffer with 3 mM $MgCl_2$ at 37 °C with constant shaking. After a 5 min preincubation, the reaction was initiated by the addition of NADPH (1 mM). At selected time intervals (0, 3, 7, 15, 25, and 45 min), aliquots were taken and subsequently placed into a 96-well plate containing cold acetonitrile with internal standard (50 ng/mL carbamazepine). Plates were then centrifuged at 3000 rcf (4 °C) for 10 min, and the supernatant was transferred to a separate 96-well plate and diluted 1:1 with water for LC/MS/MS analysis. The *in vitro* half-life ($_{1/2}$, min, Eq. 1), intrinsic clearance (CL_{int}, mL/min/kg, Eq. 2), and subsequent predicted hepatic clearance (CL_{hep}, mL/min/kg, Eq. 3) was determined employing the following equations:

(1) $T_{1/2} = \frac{Ln(2)}{K}$

where k represents the slope from linear regression analysis of the natural log percent remaining of a test compound as a function of incubation time

(2) $CL_{int} = \frac{0.693}{in\ vitro T_{1/2}}\ x\ \frac{mL\ incubation}{mg\ microsomes}\ x\ \frac{45\ mg\ microsomes}{gram\ liver}\ x\ \frac{20^{a}\ gram\ liver}{kg\ body\ wt}$

[a]scale-up factors: of 20 (human) or 45 (rat)

(3) $CL_{hep} = \frac{Q_h \cdot CL_{int}}{Q_h + CL_{int}}$

where Q_h (hepatic blood flow, mL/min/kg) is 21 (human) or 70 (rat).

LC/MS/MS Bioanalysis of Samples from Plasma Protein Binding and Intrinsic Clearance Assays:

Samples were analyzed on a Thermo Electron TSQ Quantum Ultra triple quad mass spectrometer (San Jose, CA) via electrospray ionization (ESI) with two Themo Electron Accella pumps (San Jose, CA), and a Leap Technologies CTC PAL autosampler (Carrboro, NC). Analytes were separated by gradient elution on a dual column system with two Thermo Hypersil Gold (2.1 x 30 mm, 1.9 µm) columns (San Jose, CA) thermostated at 40 °C. HPLC mobile phase A was 0.1% formic acid in water and mobile phase B was 0.1% formic acid in acetonitrile. The gradient started at 10% B after a 0.2 min hold and was linearly increased to 95% B over 0.8 min; hold at 95% B for 0.2 min; returned to 10% B in 0.1 min. The total run time was 1.3 min and the HPLC flow rate was 0.8 mL/min. While pump 1 ran the gradient method, pump 2 equilibrated the alternate column isocratically at 10% B. Compound optimization, data collection, and processing was performed using Thermo Electron's QuickQuan software (v2.3) and Xcalibur (v2.0.7 SP1).

***In vivo* DMPK experimental:**

Determination of brain to plasma ratio:

Animal care and use

All animal study procedures were approved by the Institutional Animal Care and Use Committee and were conducted in accordance with the National Institutes of Health regulations of animal care covered in Principles of Laboratory Animal Care (National Institutes of Health). All rats were fasted overnight prior to testing.

In-life phase

For determination of the brain over plasma ratio (K_p), compounds were formulated in 8% ethanol, 32% PEG400 and 60% DMSO (*v/v/v*) and administered as a single 0.2 mg/kg IV dose (1 mL/kg) to male, Sprague Dawley rats (*n* = 1) via injection into a surgically-implanted jugular vein catheter. At 15 min post dosing, blood sample was collected into chilled, K_2EDTA anticoagulant-fortified tube and immediately placed on wet ice. The blood sample was then centrifuged (1700 rcf, 5 minutes, 4 ˚C) to obtain plasma sample. At the same post-administration time point, whole

brain sample was obtained by rapid dissection, rinsed with PBS, and immediately frozen in individual tissue collection box (dry ice). All brain and plasma samples were stored at -80 ˚C until analysis by LC-MS/MS.

Sample Analysis: Concentrations in plasma and brain homogenates were quantified by liquid chromatography tandem mass spectrometry (LC-MS/MS). Whole brains were homogenized in 3 mL of 70:30 IPA:water in a mini bead beater for 3 min, and centrifuged at 3,500 g for 5 min. 5 uL of the supernatant was diluted in 15 uL of blank plasma for quantification of the analytes. Plasma samples were centrifuged at 3,500 g for 5 min. A standard curve was generated by diluting the analytes DMSO stocks with blank plasma to obtain a final concentration of 10,000 ng/ml followed by a serial dilution down to 0.5 ng/ml. Quality controls were generated by a serial dilution of the 5,000 ng/ml standard curve solution in blank plasma to obtain 3 concentrations of 500, 50, and 5 ng/ml. 20 uL of brain diluted in plasma, plasma, blank plasma, standard curve and QC samples were loaded in a V-bottom 96-well plate. 120 uL of acetonitrile containing 0.05 uM carbamazepine (internal standard) was added to each well and the plate was centrifuged at 3,500 g for 5 min. 60 uL of the supernatant of each well (protein free) was transferred to a new 96-well plate containing 60 uL of water. The plates were sealed for analysis by LC-MS/MS.

Plasma and brain tissue samples originating from *in vivo* studies were analyzed by electrospray ionization using an AB Sciex Q-TRAP 5500 (Foster City, CA) that was coupled to a Shimadzu LC-20AD pump (Columbia, MD) and a Leap Technologies CTC PAL auto-sampler (Carrboro, NC). Analytes were separated by gradient elution using a C18 column (3 x 50 mm, 3 mm; Fortis Technologies Ltd, Cheshire, UK) that was thermostated at 40 °C. HPLC mobile phase A was 0.1% formic acid in water (pH unadjusted); mobile phase B was 0.1% formic acid in acetonitrile (pH unadjusted). A 10% B gradient was held for 0.2 min and was linearly increased to 90% B over 0.8 min, with an isocratic hold for 0.5 min, before transitioning to 10% B over 0.05 min. The column was re-equilibrated (1 min) before the next sample injection. The total run time was 2.55 min, and the HPLC flow rate was 0.5 ml/min. The source temperature was set at 500 °C, and mass spectral analyses were performed using a Turbo-Ion spray source in positive ionization mode (5.0-kV spray voltage) and using multiple-reaction monitoring of transitions specific for the analytes. All data were analyzed using AB Sciex Analyst 1.5.1 software.

Brain plasma concentration ratio (K_p) was calculated by dividing brain concentration by plasma concentration for each animal. Unbound brain to unbound plasma concentration ratio ($K_{p,uu}$) is calculated using the following formula: $K_{p,uu}$ = (Brain ng/g x brain fu)/(plasma ng/ml x plasma fu).

Pharmacokinetic profiles in rats following oral single escalating doses

Single escalating oral dosing in Sprague-Dawley rats was performed at Frontage Laboratories according to their non-GLP Standard Operating Procedure and IACUC protocols. In short, compounds were formulated in 10% Tween 80 in water and dosed at 10 mg/kg. At different times, arterial blood was collected from a femoral artery catheter, and compound concentration was determined in plasma by LC-MS/MS following their non-GLP protocol. PK parameters were determined using Phoenix WinNonlin software (version 6.3).

In-vitro determination of blood-brain barrier penetration potential

Blood-brain barrier penetration was determined using MDR1-MDCK cell monolayers by Absorption Systems, following their protocol. In short, compounds were incubated at 5 mM final concentration on one side of the cell monolayer for 2 hours. Compounds concentration on either side of the monolayer was determined by LC-MS/MS and apparent permeability and efflux ratio were determined as described in Wang, Q. et al.[1]

Behavioral Manifestations of Seizure Activity To evaluate induction of behavioral manifestation of seizure activity, C57Bl/6 mice received administration of vehicle or 100 mg/kg M_1 PAM. Compounds were formulated in 30% Captisol (11) or 10% Tween 80 (2-4 and 12) (pH 7.0) at a concentration of 10 mg/mL and injected i.p. at 10 ml/kg (*n* = 3). Animals were monitored continuously and scored for behavioral manifestations of seizure activity at 5, 10, 15, and 30 min, 1, 3 and 6 h. Behavioral manifestations of seizures were scored using a modified Racine scoring system.[2] Briefly, a score of 0 represents no behavior alterations; score 1, immobility, mouth and facial movements, or facial clonus; score 2, head nodding, tail extension; score 3, forelimb clonus, repetitive movements; score 4, rearing and tonic clonic seizure; and score 5, continuous rearing and falling, severe generalized tonic clonic seizure.

Irwin Neurological Battery

The modified Irwin neurological battery was performed to assess potential autonomic or somatosensory side effects of M_1 PAMs. Mice were dosed with 56.6 or 100 mg/kg (10 ml/kg i.p., 30% Captisol, n=6) and were placed in a plexiglas test area (18” X 18” X4”) and observed from 15 min-6 hr. The mice were monitored for changes in piloerection, respiration rate, tail erection, lacrimation, salivation, defecation, motor activity, and spraddle. Changes were scored with the criteria of no change= score of 0, mild change=1 and major change =2.

Novel Object Recognition Task

Rats were habituated for 10 min for 2 consecutive days in an empty novel object recognition (NOR) arena consisting of dark-colored plexiglass box (40 × 64 × 33 cm^3). On day 3, for the experiment utilizing VU0467319 alone, rats were administered vehicle (20% HP-β-CD) or M_1 PAM (0.1-3 mg/kg, p.o.., 10 mL/kg, n = 10-18) and returned to their home cage for 90 min. For the experiment to determine the potentiation effects of VU0467319 on donepezil, VU0467319 (0.3-3 mg/kg p.o, n=11-12) or vehicle (20% HP-β-CD) was administered 60 min prior to i.p. injection of 0.3 mg/kg donepezil or vehicle (saline) and then placed back in their home cage for 30 min. Rats were then placed in the NOR arena containing two identical objects for 10 min. Following the exposure period, rats were placed back into their home cages for 24 h. The rats were then returned to the arena in which one of the previously exposed (familiar) objects was replaced by a novel object and were video recorded for 5 min while they explored the two objects. Time spent exploring each object was scored by an observer blinded to the experimental conditions and the recognition index was calculated as [(time spent exploring novel object) – (time spent exploring familiar object)]/total time exploring objects.

Contextual Freezing

On the first day the animals are weighed, marked and habituated to the experimental room for 2 hrs. The next day the animals are placed in the habituation room for 1 hour. The animals were then pretreated with VU0467319 (1-10 mg/kg p.o..10 ml/kg, n=10-12) or vehicle (20% HP-β-CD). Sixty minutes later the animals were dosed with scopolamine (0.5 mg/kg i.p. 1 ml/kg), risperidone (3 mg/kg i.p. 1 ml/kg) or vehicle (10% Tween 80). Thirty minutes later the animals are placed in the shock chambers and are presented with 3 30s 80 db 3000 hz tone terminating in a 1 s 0.5 mV shock. A 10% vanilla (1 ml) solution was used as an odor cue and the chambers were cleaned in

between animals with soapy water. Twenty four hours later the percent of time the animal spends freezing (lack of movement with the exception of breathing) is recorded.

Radial Arm Maze

The eight arm radial maze consists of eight equidistantly spaced clear plexiglas arms (69.9 X 9.8 cm) radiating from a circular central arena (internal diameter = 33.9 cm). At the end of each arm there is a food cup, the contents of which are not visible from the central platform. The task requires that the animals enter each arm to retrieve the food pellets and use spatial cues (posters mounted on the wall at the end of each arm) in the room to remember which arms of the maze they have previously entered. To provide motivation to perform the working memory task animals are food restricted to achieve 80-85% of their free-feeding weights. Maze Habituation: On the first day of habituation to the maze, reinforcements are placed near the entrance, at the mid-point, and in the food cup at the end of each arm. Each animal is placed on the maze and allowed to explore and consume the reinforcement pellets for a period of five minutes, or until all pellets are consumed. On the second day of habituation, the pellets are placed at the mid-point and in the food cup at the end of each arm. Again, the animals are allowed to explore the maze until all pellets are consumed or until five minutes have elapsed. Training begins on the third day of exposure to the maze. Maze Training: During training, one reinforcement pellet is placed in the food cup at the end of each arm. Animals are placed on the maze facing away from the experimenter, facing the same arm at the start of each trial. The timer is started and each arm entry (1 through 8) is recorded in sequence. An entry is defined as all four paws entering an arm. The animals are allowed to choose arms until all eight arms are entered and pellets are consumed, or until 30 choices are made, or until 5 minutes have elapsed. Entry into an arm previously chosen is counted as an error. If an animal fails to choose all eight arms in 5 minutes, the arms not chosen are also counted as errors. Animals are trained once a day and the training criterion is defined as two or fewer errors on two consecutive training days. Maze Testing: During testing, one reinforcement pellet is placed in the food cup at the end of each arm. The animals were pretreated with VU0467319 (1-3 mg/kg p.o..10 ml/kg, n=8) or vehicle (20% HP-β-CD). Sixty minutes later the animals were dosed with scopolamine (0.5 mg/kg i.p. 1 ml/kg) or vehicle (10% Tween 80). Animals are placed on the maze facing away from the experimenter, facing the same arm at the start of each trial. The timer is started and each arm entry (1 through 8) is recorded in sequence. An entry is defined as all four paws entering an

arm. The animals are allowed to choose arms until all eight arms are entered and pellets are consumed, or until 30 choices are made, or until 5 minutes have elapsed. Entry into an arm previously chosen is counted as an error. If an animal fails to choose all eight arms in 5 minutes, the arms not chosen are also counted as errors.

Electroencephalogram (EEG)

All animals used in telemetry studies were surgically implanted under isoflurane anesthesia with a telemetric transmitter (Data Sciences International [DSI], Minneapolis, MN) for the wireless recording of EEG, electromyographic (EMG), and motor activity. Following surgery, animals were individually housed. Prior to dosing studies, animals were recorded for 24 hrs to determine the quality of the surgery/recording and determine inclusion in the study. EEG and EMG were recorded from the home cage of each animal continuously for 24 h beginning at the onset of the light cycle on the day of each study. Following a 2 hr baseline the animals were administered VU0467319 (3-30 mg/kg p.o.) or vehicle (20% HP-β-CD) and were placed back into their homecage for the remainder of the 24 hr session. Trained observers blinded to condition scored each 10-s epoch using Neuroscore 3.0 software (DSI) to determine sleep/wake stages, including wake, NREM, or REM sleep based on accepted characteristic oscillatory patterns. The amount of time in each stage (wake, NREM, REM) in 30 min bins across a 24-h period served as primary dependent measures to determine effects of acute pharmacological challenge. Following sleep staging, quantitative EEG (qEEG) relative power spectra were computed in 1 Hz bins from 0.5 to 100 Hz using a Fast Fourier transform with a Hamming window and overlap ratio of 0.5. Relative power within each 1-Hz increment was subsequently binned by stage (wake), then averaged across a select time period to yield the state-dependent relative power spectrum for each animal and condition. Differences in spectral power between dose-effect determinations were examined in 1-h bins in a state-dependent (Wake) manner. Sleep/wake architecture and qEEG data are presented as means ± S.E.M. When possible, a repeated measures two-way analysis of variance (matching both factors) was applied. For total sleep and individual spectra analysis, one way ANOVA was utilized. In all cases, main effects were followed by Dunnett's or Bonferroni's multiple comparison test (see Table S2). GraphPad Prism was used for all graphing and statistical applications.

Amphetamine or MK-801-induced Hyperlocomotion

Adult male Sprague-Dawley rats placed into the activity chambers (Med Associates). Thirty minutes after placement in the chambers, the rats were administered VU0467319 (10-56.6 mg/kg p.o. 10 ml/kg.) or vehicle. Sixty minutes later the rats were injected with amphetamine, MK-801, or vehicle and placed back in the activity chambers for 60 minutes. The data f were analyzed by a between-group 2 way analysis of variance for compound dose and time. Each dose group was compared with the vehicle control group. The calculations were performed and graphed using GraphPad Prism (version 4.03, GraphPad, La Jolla, CA).

Spontaneous Locomotor Activity

Adult male Sprague-Dawley rats were administered VU0467319 (3-10 mg/kg p.o.10 ml/kg) or vehicle and were placed back in their homecage. Sixty minutes later the animals were placed into the activity chambers (Med Associates) for thirty minutes.

Rotarod

On the first day, the animals were placed on the rotarod apparatus which was rotating at a speed of 20 rpm. If the rats fall of the bar, the animals were placed back on the apparatus for a total of 2 min. On the day of testing, adult male rats were pretreated with VU0467319 (3-10 mg/kg p.o. 10 ml/kg) 60 minutes before being placed on the rotarod apparatus. Testing consisted of accelerating rotarod from 4 to 40 rpm over 5 min. The time and final speed when the rats fall off of the apparatus was recorded.

Supplemental field electrophysiology methods

Recording pipettes were constructed from thin-walled borosilicate capillary glass tubing (I.D.=1.17 mm, O.D. 1.50 mm; Warner Instruments, Hamden, CT), pulled with a horizontal pipette puller (P-97 Sutter Instrument Co., Novata, CA) to a resistance of 1-3MΩ when filled with ACSF. Layer II/III was visualized using an Olympus BX50WI upright microscope (Olympus, Lake Success, NY) microscope according to landmarks illustrated in the Allen mouse brain atlas (Lein et al, 2007) and the recording electrode was laterally placed approximately 200µM away from layer 2/3 into layer V so that the recording and stimulating electrodes were parallel to each other.

Inputoutput curves were generated to determine the stimulus intensity that produced approximately 70% of the maximum fEPSP slope before each experiment, which was then used as the baseline stimulation. Similarly, the recording electrode for ofEPSP was placed in layer V and an input-output curve was generated to produce approximately 70% of the maximal ofEPSP slope. Data were digitized using a Multiclamp 700B, using a sampling rate of 20,000kHz and were filtered at 0.5kHz, with a Digidata 1322A, pClamp 9.2 and Clampex 10.6.2 software (Molecular Devices) running on a Dell PC (Round Rock, TX). Offline data analysis to calculate fEPSP slope or ofEPSP slope was performed using Clampfit 10.2 (Molecular Devices).

General Methods

All NMR spectra were recorded on a 400 MHz AMX Bruker NMR spectrometer. ^{1}H and ^{13}C chemical shifts are reported in δ values in ppm downfield with the deuterated solvent as the internal standard. Data are reported as follows: chemical shift, multiplicity (s = singlet, d = doublet, t = triplet, q = quartet, b = broad, m = multiplet), integration, coupling constant (Hz). Low resolution mass spectra were obtained on an Agilent 6120/6150 or Waters QDa (Performance) SQ MS with ESI source. *Method A (Agilent 6120/6150):* MS parameters were as follows: fragmentor: 70, capillary voltage: 3000 V, nebulizer pressure: 30 psig, drying gas flow: 13 L/min, drying gas temperature: 350 ºC. Samples were introduced via an Agilent 1290 UHPLC comprised of a G4220A binary pump, G4226A ALS, G1316C TCC, and G4212A DAD with ULD flow cell. UV absorption was generally observed at 215 nm and 254 nm with a 4 nm bandwidth. Column: Waters Acquity BEH C18, 1.0 x 50 mm, 1.7 um. Gradient conditions: 5% to 95% CH_3CN in H_2O (0.1% TFA) over 1.4 min, hold at 95% CH_3CN for 0.1 min, 0.5 mL/min, 55 ºC. *Method B (Agilent 6120/6150):* MS parameters were as follows: fragmentor: 100, capillary voltage: 3000 V, nebulizer pressure: 40 psig, drying gas flow: 11 L/min, drying gas temperature: 350 ºC. Samples were introduced via an Agilent 1200 HPLC comprised of a degasser, G1312A binary pump, G1367B HP-ALS, G1316A TCC, G1315D DAD, and a Varian 380 ELSD (if applicable). UV absorption was generally observed at 215 nm and 254 nm with a 4 nm bandwidth. Column: Thermo Accucore C18, 2.1 x 30 mm, 2.6 um. Gradient conditions: 7% to 95% CH_3CN in H_2O (0.1% TFA) over 1.6 min, hold at 95% CH_3CN for 0.35 min, 1.5 mL/min, 45 ºC. *Method C (Waters QDa (Performance)*

SQ MS): MS parameters were as follows: cone voltage: 15 V, capillary voltage: 0.8 kV, probe temperature: 600° C. Samples were introduced via an Acquity I-Class PLUS UPLC comprised of a BSM, FL-SM, CH-A, and PDA. UV absorption was generally observed at 215 nm and 254 nm; 4 nm bandwidth. Column: Phenomenex EVO C18, 1.0 x 50 mm, 1.7 um. Column temperature: 55° C. Flow rate: 0.4 mL/min. Default gradient: 5% to 95% CH_3CN (0.05% TFA) in H_2O (0.05% TFA) over 1.4 min (curve 6), hold at 95% CH_3CN for 0.1 min. "Polar" (2% to 70% CH_3CN (0.05% TFA) in H_2O (0.05% TFA) over 0.8 min (curve 6), transition to 95% CH_3CN over 0.1 min (curve 6), hold at 95% CH_3CN for 0.6 min.) and "Non-Polar" (40% to 95% CH_3CN (0.05% TFA) in H_2O (0.05% TFA) over 1.4 min (curve 6), hold at 95% CH_3CN for 0.1 min.) gradients were also available. *Method D (Waters QDa (Performance) SQ MS):* MS parameters were as follows: cone voltage: 15 V, capillary voltage: 0.8 kV, probe temperature: 600° C. Samples were introduced via an Acquity I-Class PLUS UPLC comprised of a BSM, FL-SM, CH-A, and PDA. UV absorption was generally observed at 215 nm and 254 nm with a 4 nm bandwidth. Column: Phenomenex EVO C18, 1.0 x 50 mm, 1.7 um. Column temperature: 55° C. Flow rate: 0.4 mL/min. Default gradient: 5% to 95% CH_3CN in H_2O (5 mM NH_4HCO_3) over 1.4 min (curve 6), hold at 95% CH_3CN for 0.1 min. "Polar" (2% to 70% CH_3CN in H_2O (5 mM NH_4HCO_3) over 0.8 min (curve 6), transition to 95% CH_3CN over 0.1 min (curve 6), hold at 95% CH_3CN for 0.6 min.) and "Non-Polar" (40% to 95% CH_3CN in H_2O (5 mM NH_4HCO_3) over 1.4 min (curve 6), hold at 95% CH_3CN for 0.1 min.) gradients were also available. High resolution mass spectra were obtained on an Agilent 6540 UHD Q-TOF with ESI source. MS parameters were as follows: fragmentor: 150, capillary voltage: 3500 V, nebulizer pressure: 60 psig, drying gas flow: 13 L/min, drying gas temperature: 275 ºC. Samples were introduced via an Agilent 1200 UHPLC comprised of a G4220A binary pump, G4226A 3 ALS, G1316C TCC, and G4212A DAD with ULD flow cell. UV absorption was observed at 215 nm and 254 nm with a 4 nm bandwidth. Column: Agilent Zorbax Extend C18, 1.8 μm, 2.1 x 50 mm. Gradient conditions: 5% to 95% CH_3CN in H_2O (0.1% formic acid) over 1 min, hold at 95% CH_3CN for 0.1 min, 0.5 mL/min, 40 ºC. For compounds that were purified on a Gilson preparative reversed-phase HPLC, the system comprised of a 333 aqueous pump with solvent selection valve, 334 organic pump, GX 271 or GX-281 liquid hander, two column switching valves, and a 155 UV detector. UV wavelength for fraction collection was user-defined, with absorbance at 254 nm always monitored. Method 1: Phenomenex Axia-packed Luna C18, 30 x 50 mm, 5 μm column. Mobile phase: CH_3CN in H_2O (0.1% TFA). Gradient

conditions: 0.75 min equilibration, followed by user defined gradient (starting organic percentage, ending organic percentage, duration), hold at 95% CH_3CN in H_2O (0.1% TFA) for 1 min, 50 mL/min, 23 ºC. Method 2: Phenomenex Axia packed Gemini C18, 50 x 250 mm, 10 um column. Mobile phase: CH_3CN in H_2O (0.1% TFA). Gradient conditions: 7 min equilibration, followed by user defined gradient (starting organic percentage, ending organic percentage, duration), hold at 95% CH_3CN in H_2O (0.1% TFA) for 7 min, 120 mL/min, 23 ºC. Solvents for extraction, washing and chromatography were HPLC grade. All compounds are >95% purity by HPLC.

Synthetic Procedures:

Discovery procedures for the synthesis of VU0467319 (16, VU319/ACP-319)

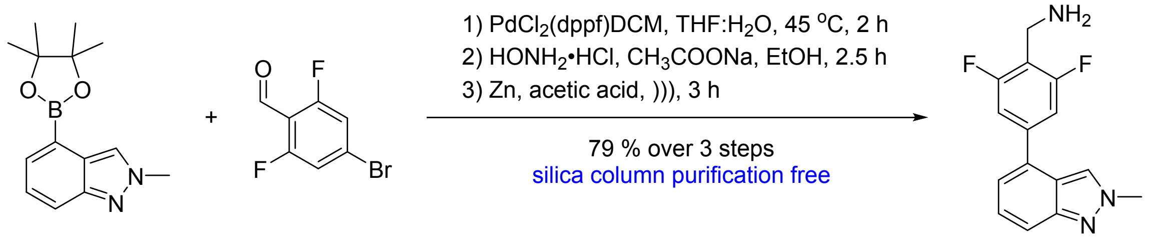

17 **18** **19**

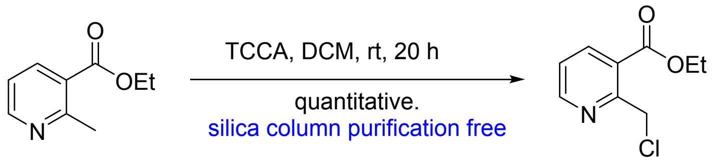

20 **21**

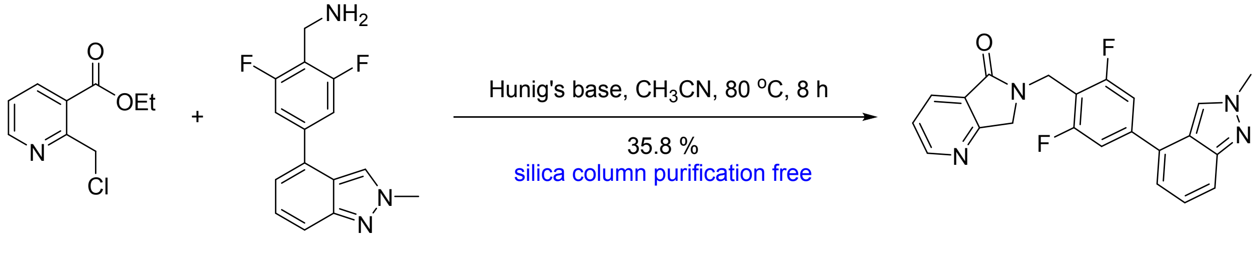

21 **19** **16**

Step A. Synthesis of (2,6-difluoro-4-(2-methyl-2*H*-indazol-4-yl)phenyl)methanamine (19).

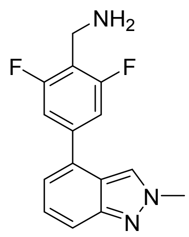

19

Step 1. 2,6-Difluoro-4-(2-methyl-2*H*-indazol-4-yl)benzaldehyde. To a solution of 2,6-difluoro-4-bromobenaldehyde **18** (13.2 g, 60 mmol) in THF (250 mL), pinacol borane (16.5 g, 64 mmol), $PdCl_2$(dppf)DCM (2.5 g, 3 mmol), Cs_2CO_3 (58.6 g, 180 mmol), and H_2O (40 mL) were added. The reaction mixture was then purged with N_2 and stirred at 45 °C for 2 h. Upon completion, the reaction mixture was cooled to room temperature and diluted with H_2O. The desired product was crashed out of solution over time and collected by vacuum filtration. The obtained product was washed with H_2O and diethyl ether before dried *in vacuo* to provide the crude mixture of the title product (18.02 g of crude was obtained, and a quantitative yield 16.3 g was assumed). This was used for the next step without further purification. LCMS ES-MS $[M+H]^+$ = 272.9.

Step 2. 2,6-difluoro-4-(2-methyl-2*H*-indazol-4-yl)benzaldehyde oxime. To a solution of 2,6-difluoro-4-(2-methyl-2*H*-indazol-4-yl)benzaldehyde (16.3 g, 60 mmol) in EtOH (380 mL), hydroxylamine hydrochloride (6.3 g, 90 mmol) and sodium acetate (7.4 g, 90 mmol) were added. This heterogeneous reaction mixture was stirred at room temperature for 2.5 h. Upon completion, the reaction mixture was filtered to collect the title product and washed with EtOH. The combined filtrate was then concentrated and filtered to collect the remaining products (21.73 g of crude was obtained, and a quantitative yield 17.2 g was assumed). This was used for the next step without further purification. LCMS ES-MS $[M+H]^+$ = 287.9.

Step 3. (2,6-difluoro-4-(2-methyl-2*H*-indazol-4-yl)phenyl)methanamine (19). 2,6-difluoro-4-(2-methyl-2*H*-indazol-4-yl)benzaldehyde oxime (17.2 g, 60 mmol) and glacial acetic acid (380 mL) were mixed in a reaction flask. To this reaction mixture, Zn dust (20 g) was slowly added in 4 portions over 4 min. The reaction mixture was then stirred at room temperature for 2.5 h while vented under a nitrogen atmosphere. The reaction was stalled after this time. Therefore, additional Zn dust (20 g) was slowly added and sonicated for 3 h. After this time, the reaction went to completion under the organo-sonochemistry condition. The reaction mixture was then passed through a pad of Celite and washed with DCM. Combined organics were concentrated using an azeotrope of toluene and MeOH. The crude mixture was then dissolved in 100 mL of H_2O, filtered, and washed with H_2O. Combined aqueous filtrate was washed with DCM. The aqueous filtrate was then diluted with DCM and neutralized with K_2CO_3. Free-based product was extracted with DCM, dried over with Na_2SO_4, filtered, and concentrated to give the title product (13.05 g, 79% over 3 steps). LCMS ES-MS $[M+H]^+$ = 273.9. ^1H NMR (400 Mhz, $CDCl_3$) δ 8.00 (s, 1H), 7.69 (d, *J* = 8.7 Hz, 1H), 7.32 (dd, *J* = 8.6, 6.9 Hz, 1H), 7.16 (m, 2H), 7.1 (d, *J* = 6.9 Hz, 1H), 4.22 (s, 3H), 3.96 (s, 2H), 1.57 (br s, 2H).

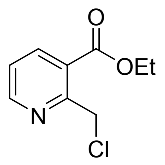

21

Step B. Synthesis of ethyl 2-(chloromethyl)nicotinate (21). To a solution of 2-methylnicotinate **20** (8260 mg, 50 mmol) in DCM (200 mL), TCCA (17.43 g, 75 mmol) was added and stirred for 20 h at room temperature. Upon completion, sat. aq. NH_4Cl solution was added to quench the reaction. The organic layer was then extracted with DCM, dried over Na_2SO_4, filtered, and concentrated *in vacuo* to afford the crude mixture of title product (11.53 g of crude was obtained, and a quantitative yield 9.98 g was assumed). This was used for the next step without further purification. LCMS ES-MS $[M+H]^+$ = 200.4.

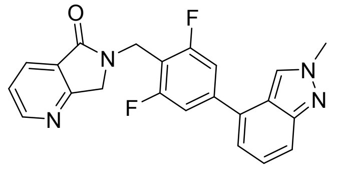

16

Step C. Synthesis of 6-(2,6-difluoro-4-(2-methyl-2*H*-indazol-4-yl)benzyl)-6,7-dihydro-5*H*-pyrrolo[3,4-*b*]pyridin-5-one (VU0467319, 16). To a solution of ethyl 2-(chloromethyl)nicotinate **21** (10 g, 50.1 mmol) in CH_3CN (150 mL), Hunig's base (7.95 mL, 45.5 mmol) was added followed by (2,6-difluoro-4-(2-methyl-2*H*-indazol-4-yl)phenyl)methanamine **19** (12.44 g, 45.5 mmol) in CH_3CN (100 mL). The reaction mixture was stirred at 80 °C for 8 h. The reaction mixture was cooled to room temperature and then frozen in a dry ice acetone bath. The reaction mixture was then slowly warmed to room temperature. The title product crashed out during this process and was collected by filtration. Obtained product was washed with cold H_2O and CH_3CN to afford the pure product as a white solid (7.0 g, 35.8%). LCMS ES-MS $[M+H]^+$ = 391.2. ^{1}H NMR (400 MHz, $CDCl_3$) δ 8.71 (dd, *J* = 5.0, 1.6 Hz, 1H), 8.14 (dd, *J* = 7.7, 1.7 Hz, 1H), 8.03 (s, 1H), 7.73 (dt, *J* = 8.7, 1.0 Hz, 1H), 7.39 (dd, *J* = 7.7, 5.0 Hz, 1H), 7.35 (dd, *J* = 8.7, 7.0 Hz, 1H), 7.28 – 7.22 (m, 2H), 7.16 – 7.12 (m, 1H), 5.01 (s, 2H), 4.45 (s, 2H), 4.25 (s, 3H); ^{13}C NMR (101 MHz, $CDCl_3$) δ 166.3, 163.2 (d, *J* = 8.8 Hz), 162.1, 160.7 (d, *J* = 8.8 Hz), 152.6, 149.5, 143.4 (t, *J* = 10.1 Hz), 132.2, 131.5 (t, *J* = 2.5 Hz), 126.3, 126.2, 123.3 (d, *J* = 8.5 Hz), 121.3, 120.7, 118.0, 111.4 – 110.6 (m, 4C), 51.2, 40.7, 33.8 (t, *J* = 3.5 Hz); MP: 203.9 – 204.2; HRMS (TOF, ES$^+$): $[M + H]^+$ calcd for $C_{22}H_{16}F_2N_4O$, 391.1365; found, 391.1365.

HPLC

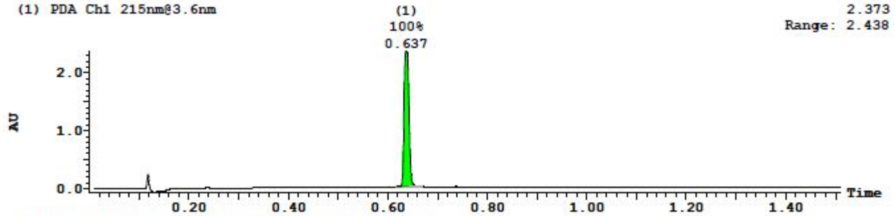

Peak Number	Time	Area %Total	BPM	Mass Found
1	0.637	100.00	391	390.0000

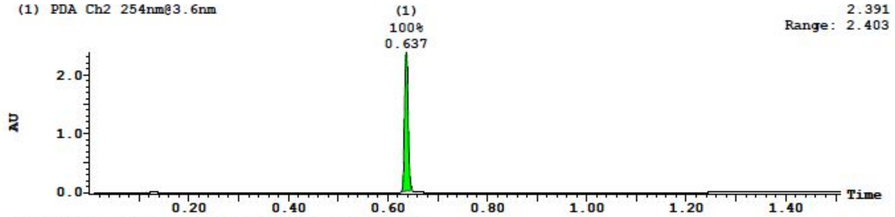

Peak Number	Time	Area %Total	BPM	Mass Found
1	0.637	100.00	391	390.0000

NMR Spectra

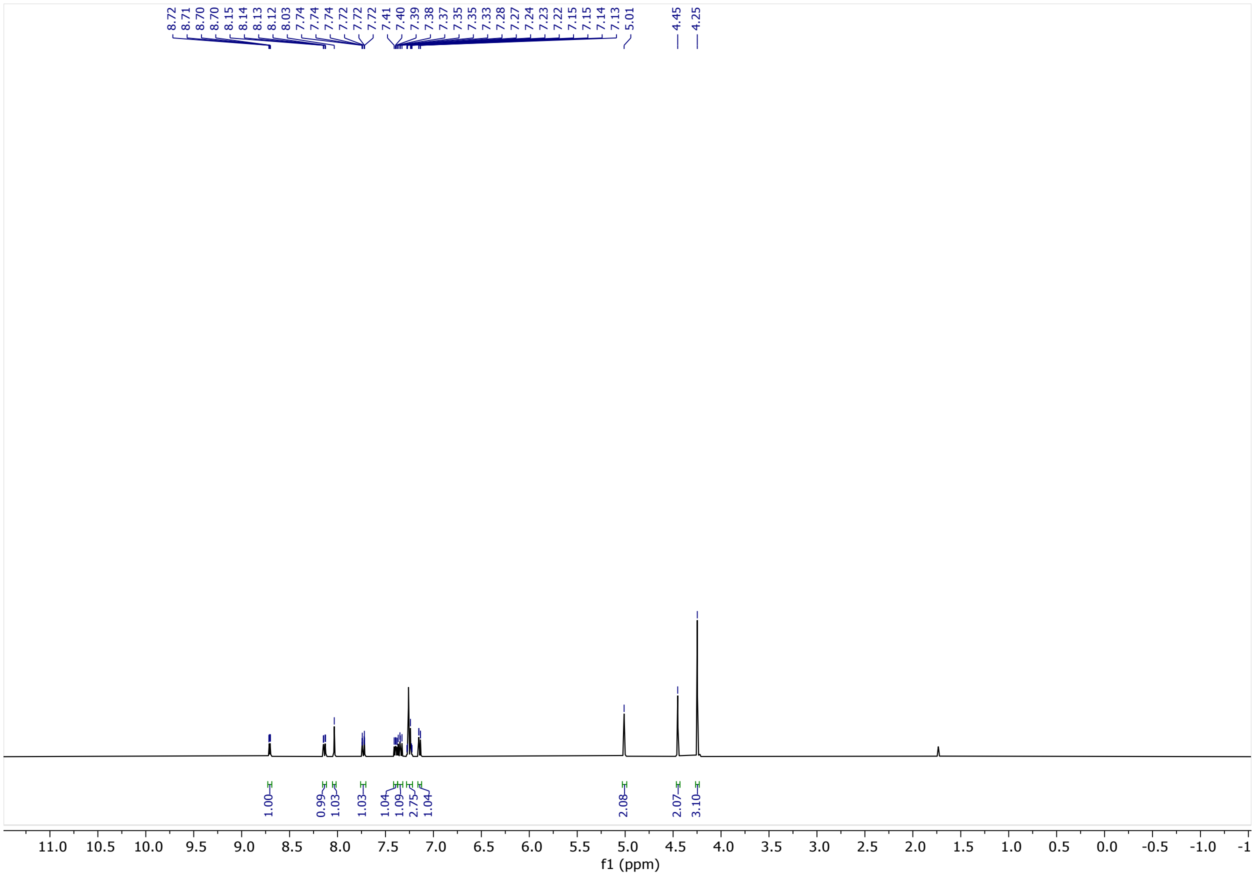

^{1}H NMR (400 MHz, $CDCl_3$) δ 8.71 (dd, J = 5.0, 1.6 Hz, 1H), 8.14 (dd, J = 7.7, 1.7 Hz, 1H), 8.03 (s, 1H), 7.73 (dt, J = 8.7, 1.0 Hz, 1H), 7.39 (dd, J = 7.7, 5.0 Hz, 1H), 7.35 (dd, J = 8.7, 7.0 Hz, 1H), 7.28 – 7.22 (m, 2H), 7.16 – 7.12 (m, 1H), 5.01 (s, 2H), 4.45 (s, 2H), 4.25 (s, 3H).

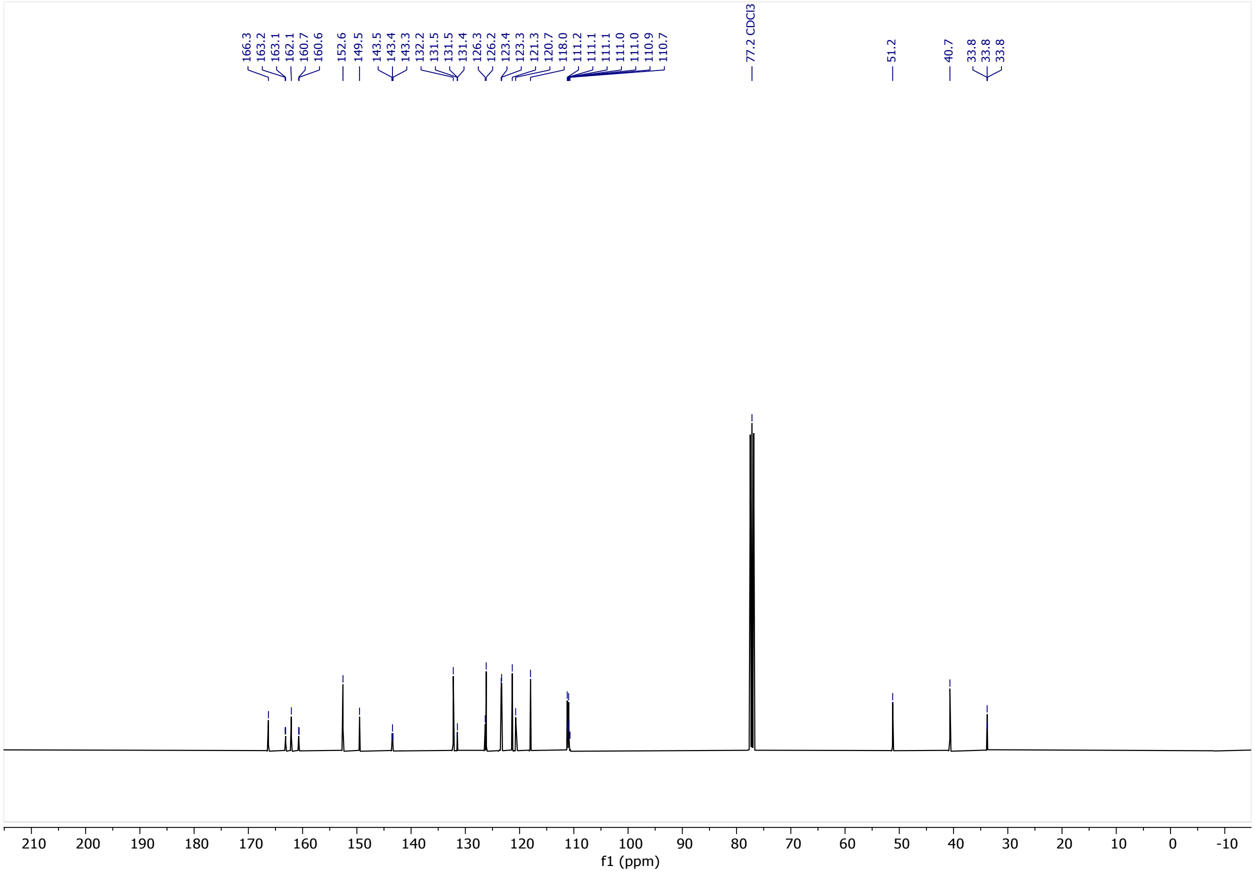

^{13}C NMR (101 MHz, $CDCl_3$) δ 166.3, 163.2 (d, J = 8.8 Hz), 162.1, 160.7 (d, J = 8.8 Hz), 152.6, 149.5, 143.4 (t, J = 10.1 Hz), 132.2, 131.5 (t, J = 2.5 Hz), 126.3, 126.2, 123.3 (d, J = 8.5 Hz), 121.3, 120.7, 118.0, 111.4 – 110.6 (m, 4C), 51.2, 40.7, 33.8 (t, J = 3.5 Hz).

Discovery procedures for the synthesis of VU0481424 (Metabolite D, 26)

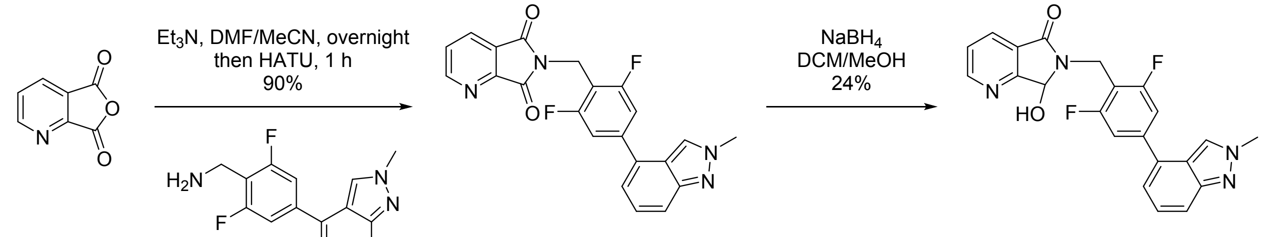

Step A. Synthesis of 6-(2,6-difluoro-4-(2-methyl-2*H*-indazol-4-yl)benzyl)-5*H*-pyrrolo[3,4-*b*]pyridine-5,7(6*H*)-dione Quinolinic anhydride (300 mg, 2.01 mmol) and (2,6-difluoro-4-(2-methyl-2H-indazol-4-yl)phenyl)methanamine (550 mg, 2.01 mmol) were dissolved in MeCN (4 mL) and DMF (3 mL). The reaction mixture was then stirred at room temperature for 30 min. To this reaction mixture, Et_3N (1.12 mL, 8.05 mmol) was added and stirred overnight at room temperature. HATU (1530 mg, 4.02 mmol) was then added and stirred at room temperature for 1 h. Upon completion, the reaction mixture was diluted with EtOAc (50 mL), washed with H_2O (10 mL x 3), and dried over Na_2SO_4. The combined organics were concentrated under reduced pressure. The crude was then purified by flash column chromatography eluting 0-100% EtOAc in hexanes to give the title product (738.5 mg, 90%). LCMS ES-MS $[M+H]^+$ = 405.

Step B. Synthesis of 6-(2,6-difluoro-4-(2-methyl-2*H*-indazol-4-yl)benzyl)-7-hydroxy-6,7-dihydro-5*H*-pyrrolo[3,4-*b*]pyridin-5-one To a solution of 6-(2,6-difluoro-4-(2-methyl-2*H*-indazol-4-yl)benzyl)-5*H*-pyrrolo[3,4-*b*]pyridine-5,7(6*H*)-dione (700 mg, 1.73 mmol) in DCM (12 mL), $NaBH_4$ (328 mg, 8.66 mmol) was added and stirred at room temperature for 3 min. To this reaction mixture, MeOH (12 mL) was added dropwise. The reaction mixture was then stirred at room temperature for 2 h. Upon completion, the reaction mixture was quenched with acetone (5 mL) and stirred for 10 min. The reaction mixture was then diluted with H_2O (20 mL) and extracted with CH_2Cl_2 (100 mL x 3). The combined extracts were dried over Na_2SO_4, filtered, and concentrated to dryness. The crude was then purified by flash column chromatography eluting 0-20% MeOH in DCM to give the title product (172.3 mg, 24%). ^1H NMR (400 MHz, $CDCl_3$) δ 8.76 (dd, *J* = 5.1, 1.6 Hz, 1H), 8.18 (dd, *J* = 7.7, 1.5 Hz, 1H), 7.99 (s, 1H), 7.70 (d, *J* = 8.7 Hz, 1H), 7.51 (dd, *J* = 7.7, 5.0 Hz, 1H), 7.32 (dd, *J* = 8.7, 6.9 Hz, 1H), 7.23 – 7.16 (m, 2H), 7.09 (d, *J* = 6.9 Hz, 1H), 6.02 (s, 1H), 5.33 (d, *J* = 14.7 Hz, 1H), 4.77 (d, *J* = 14.6 Hz, 1H), 4.22 (s, 3H). *OH is not shown; ^{13}C NMR (101 MHz, $CDCl_3$) δ 164.6, 164.0, 163.3 (d, *J* = 8.8 Hz), 160.8 (d, *J* = 9.2 Hz), 152.1, 149.5, 143.2 (t, *J* = 10.1 Hz), 133.0, 131.5 (t, *J* = 2.3 Hz), 126.3, 126.1, 125.0, 123.3, 121.3, 120.7, 117.9, 111.6 – 110.7 (m, 3C), 80.1, 40.6, 30.5 (t, *J* = 3.7 Hz); MP: 184.4 – 185.9 °C; HRMS (TOF, ES^+): $[M + H]^+$ calcd for $C_{22}H_{16}F_2N_4O_2$, 407.1314; found, 407.1317. 6-(2,6-Difluoro-4-(2-methyl-2*H*-indazol-4-yl)benzyl)-5-hydroxy-5,6-dihydro-7*H*-pyrrolo[3,4-*b*]pyridin-7-one was also isolated as a byproduct (51.5 mg). LCMS ES-MS $[M+H]^+$ = 407.

HPLC

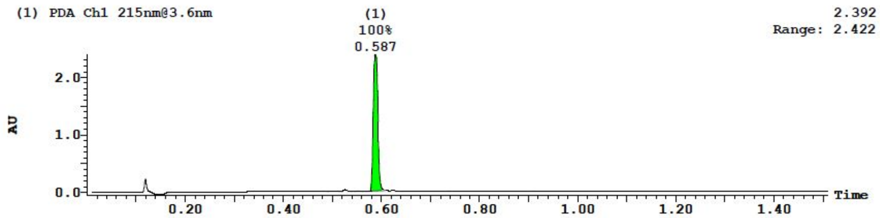

Peak Number	Time	Area %Total	BPM	Mass Found
1	0.587	100.00	407	406.3900

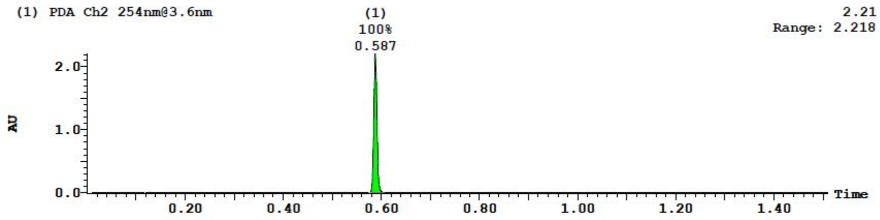

NMR Spectra

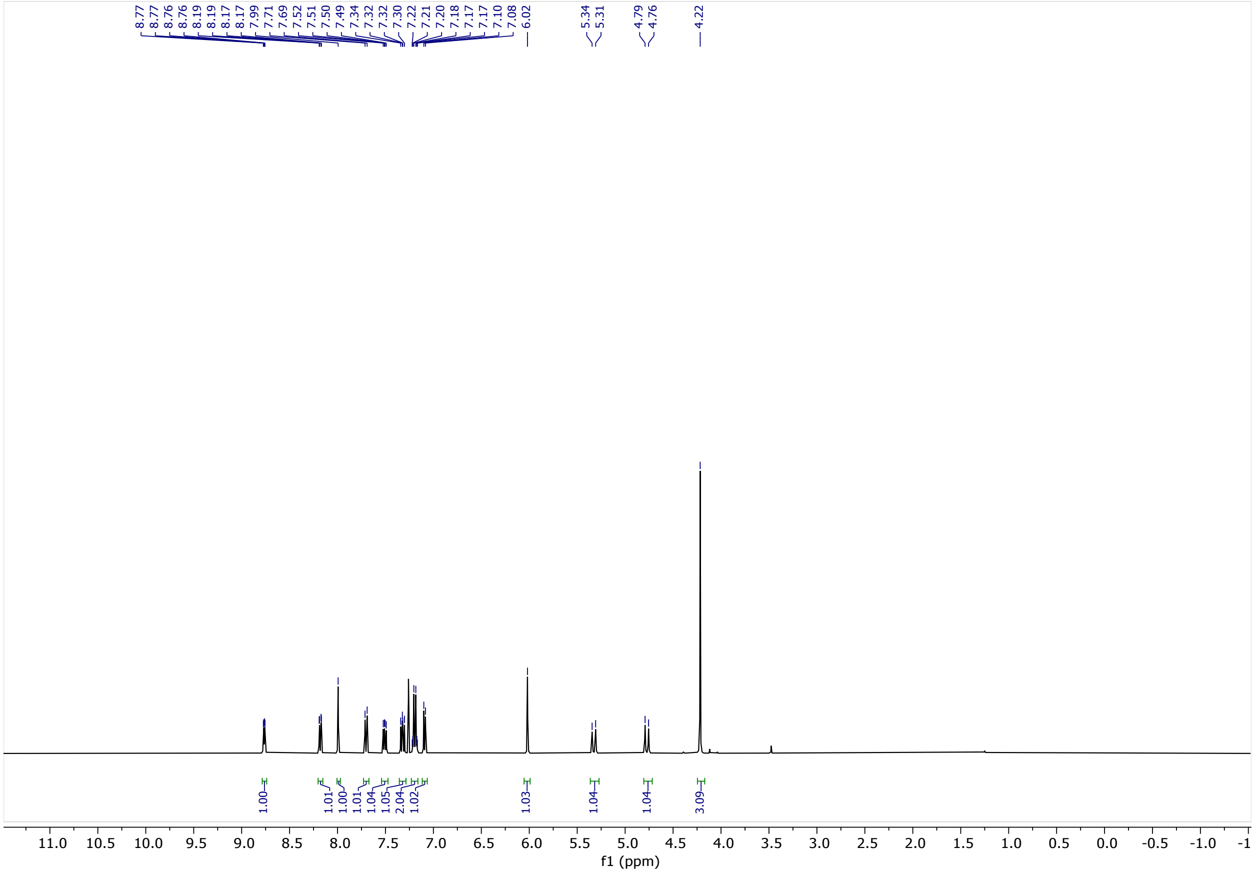

^{1}H NMR (400 MHz, $CDCl_3$) δ 8.76 (dd, J = 5.1, 1.6 Hz, 1H), 8.18 (dd, J = 7.7, 1.5 Hz, 1H), 7.99 (s, 1H), 7.70 (d, J = 8.7 Hz, 1H), 7.51 (dd, J = 7.7, 5.0 Hz, 1H), 7.32 (dd, J = 8.7, 6.9 Hz, 1H), 7.23 – 7.16 (m, 2H), 7.09 (d, J = 6.9 Hz, 1H), 6.02 (s, 1H), 5.33 (d, J = 14.7 Hz, 1H), 4.77 (d, J = 14.6 Hz, 1H), 4.22 (s, 3H). *OH is not shown.

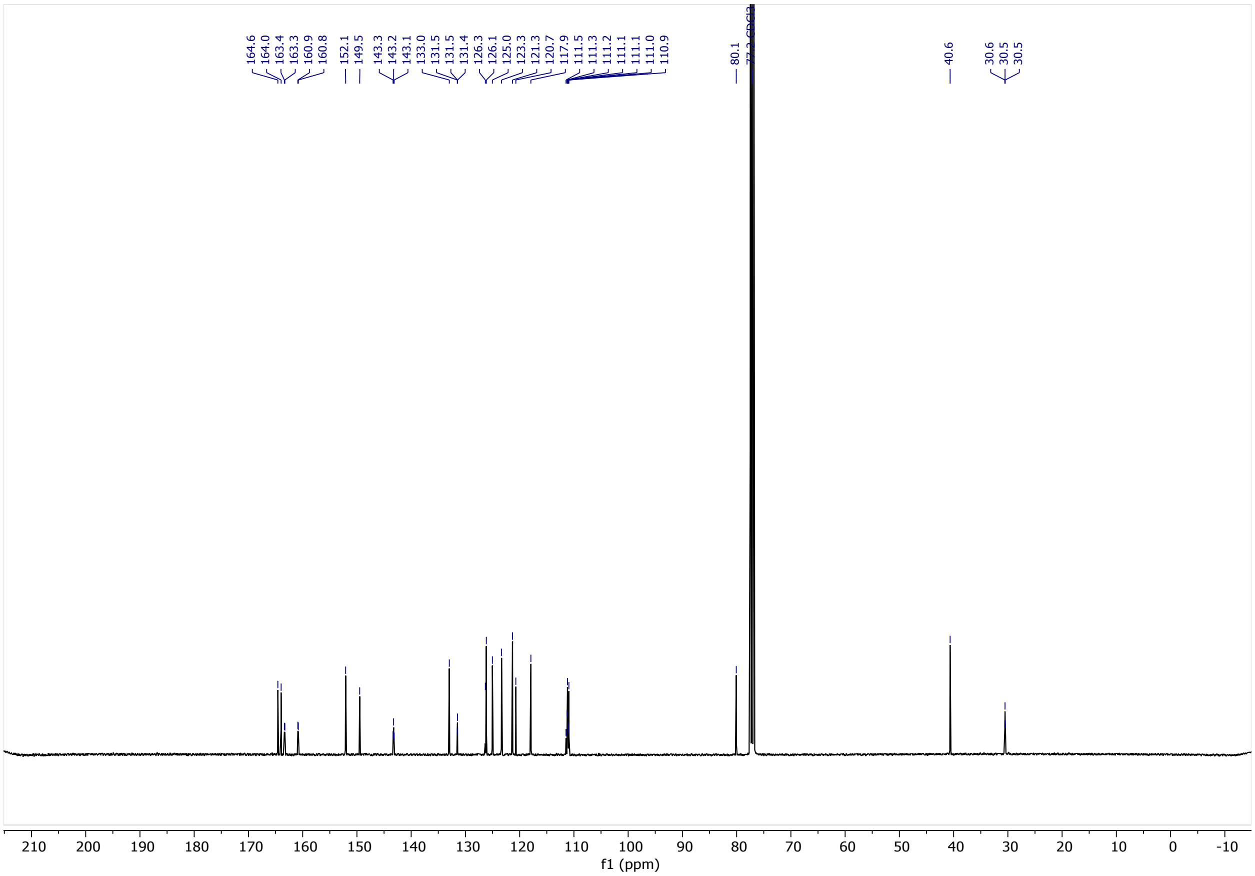

^{13}C NMR (101 MHz, $CDCl_3$) δ 164.6, 164.0, 163.3 (d, J = 8.8 Hz), 160.8 (d, J = 9.2 Hz), 152.1, 149.5, 143.2 (t, J = 10.1 Hz), 133.0, 131.5 (t, J = 2.3 Hz), 126.3, 126.1, 125.0, 123.3, 121.3, 120.7, 117.9, 111.6 – 110.7 (m, 3C), 80.1, 40.6, 30.5 (t, J = 3.7 Hz).

NOE spectra of VU0481424

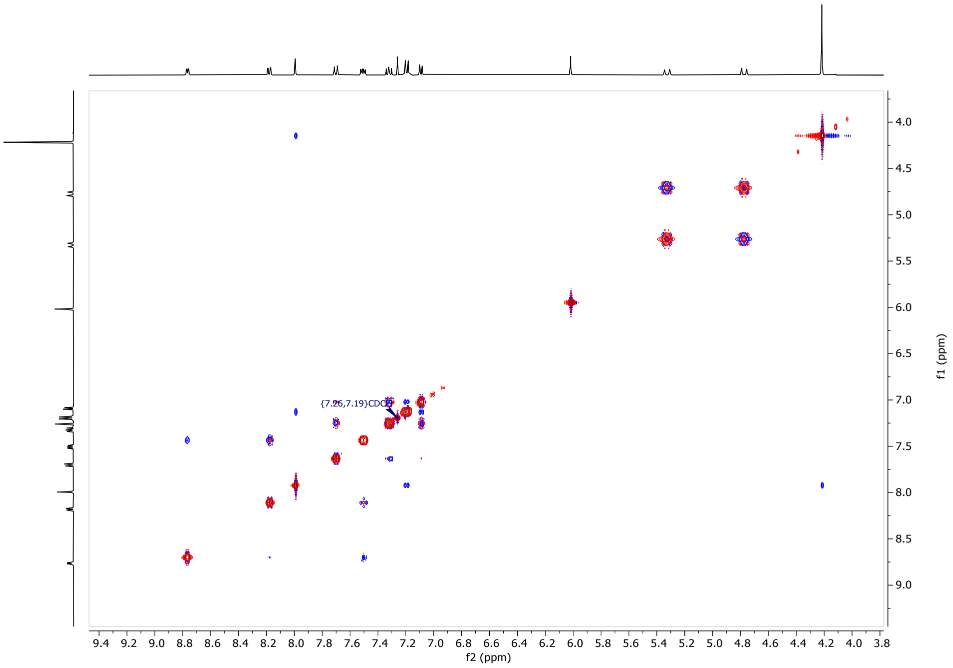

NOE spectra of byproduct

a: product, b: byproduct

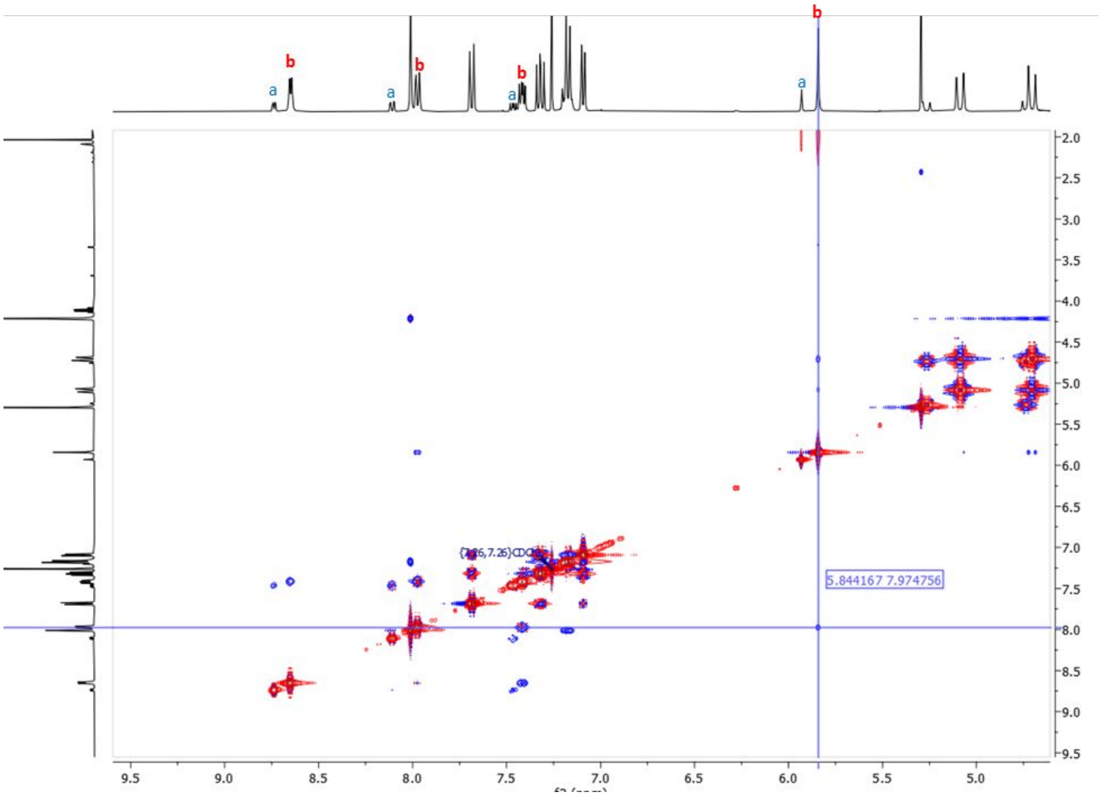

Supplemental Figures

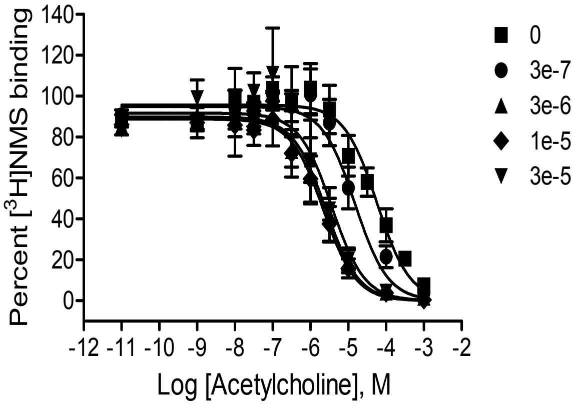

Figure S1. The M_1 PAM VU319 shifts the K_i of ACh to the left at rat M_1. The ACh affinity was shifted with a calculated α value of 59.0±42.6 (Mean ± SEM, n=2-7 replicates performed in duplicate).

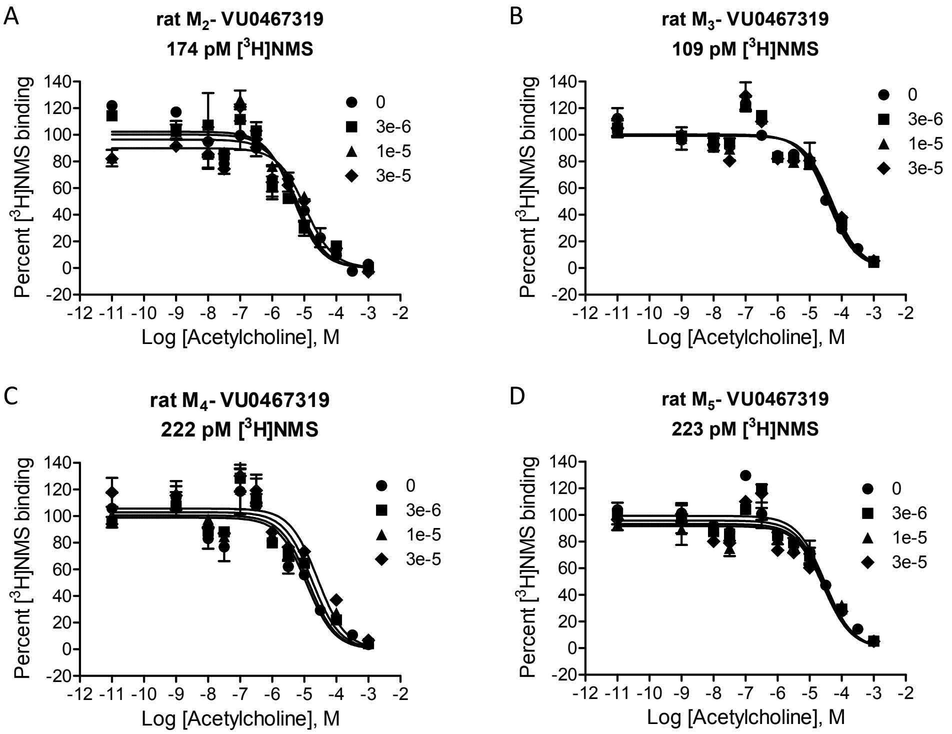

Figure S2.VU319 does not shift the affinity of either [^{3}H] NMS or ACh at rat M_2, M_3, M_4 or M_5 receptors. Values represent one experiment performed in duplicate

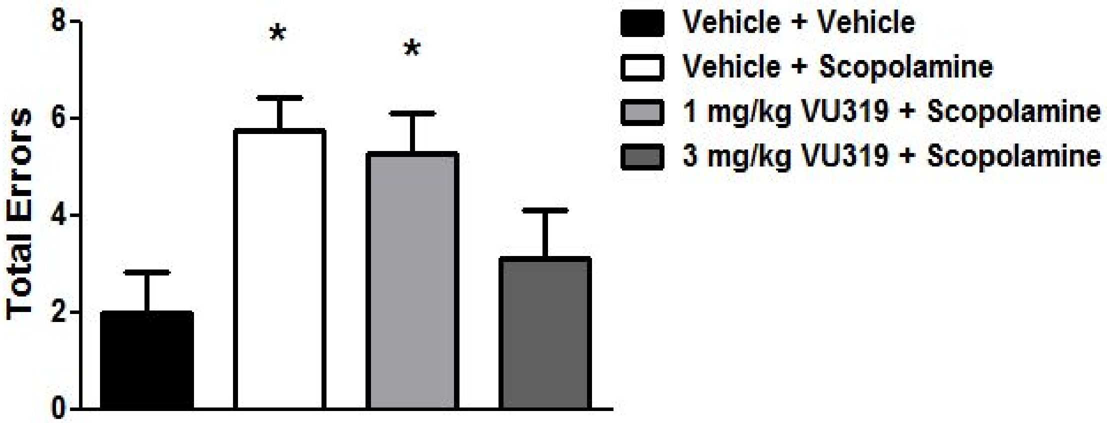

Figure S3. VU319 (PO) dose-dependently reversed scopolamine (IP)-induced deficits in the eight arm RAM task as shown by decreased total errors committed in rats.

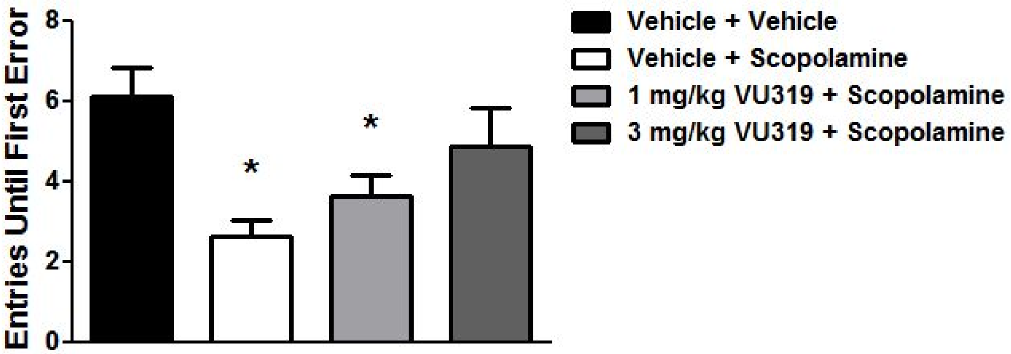

Figure S4. VU319 (PO) dose-dependently reversed scopolamine (IP)-induced deficits in the eight arm RAM task as shown by increased number of correct entries prior to first error committed in rats.

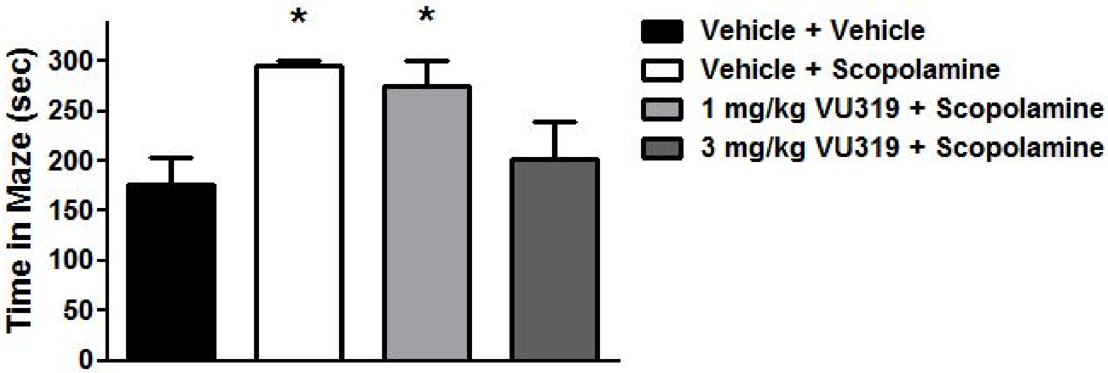

Figure S5. VU319 (PO) dose-dependently reversed scopolamine (IP)-induced deficits in the eight arm RAM task as shown by decreased total time to complete the maze in rats.

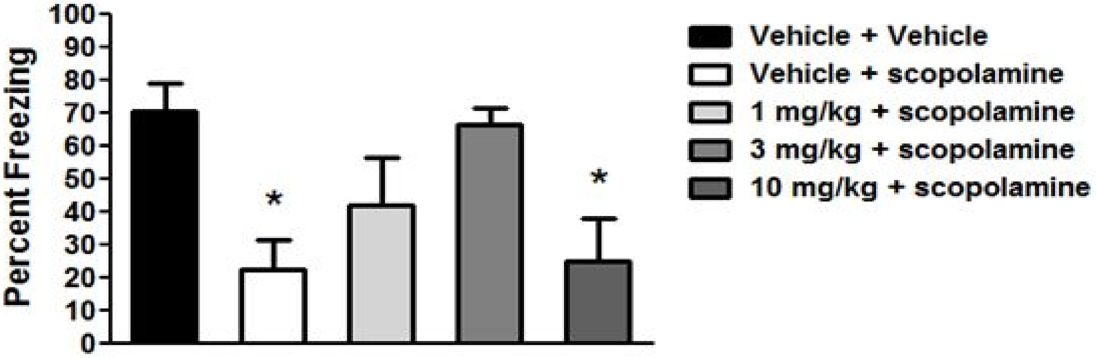

Figure S6. VU319 (PO) reversed scopolamine (IP)-induced deficits in contextual fear conditioning in rats.

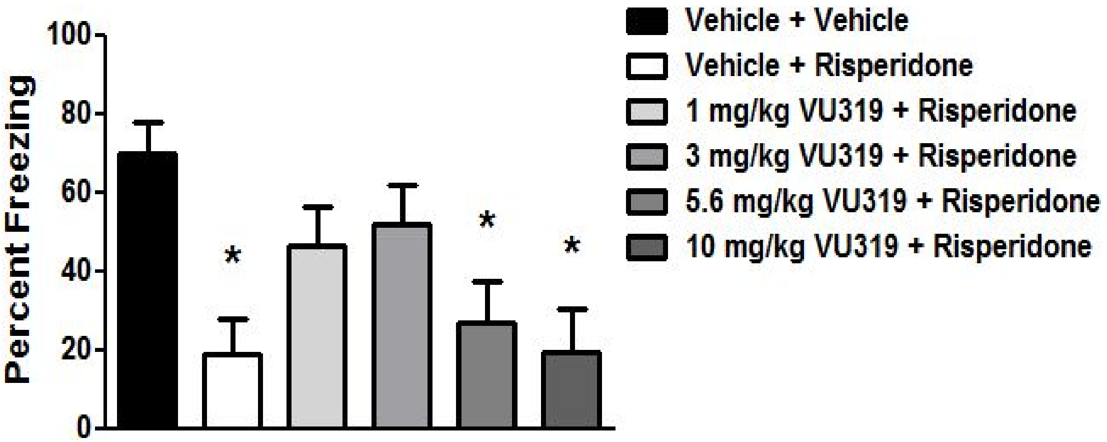

Figure S7. VU319 (PO) produced a U-shaped reversal of risperidone (IP)-induced deficits in contextual fear conditioning as shown by changes in total freezing behavior in rats.

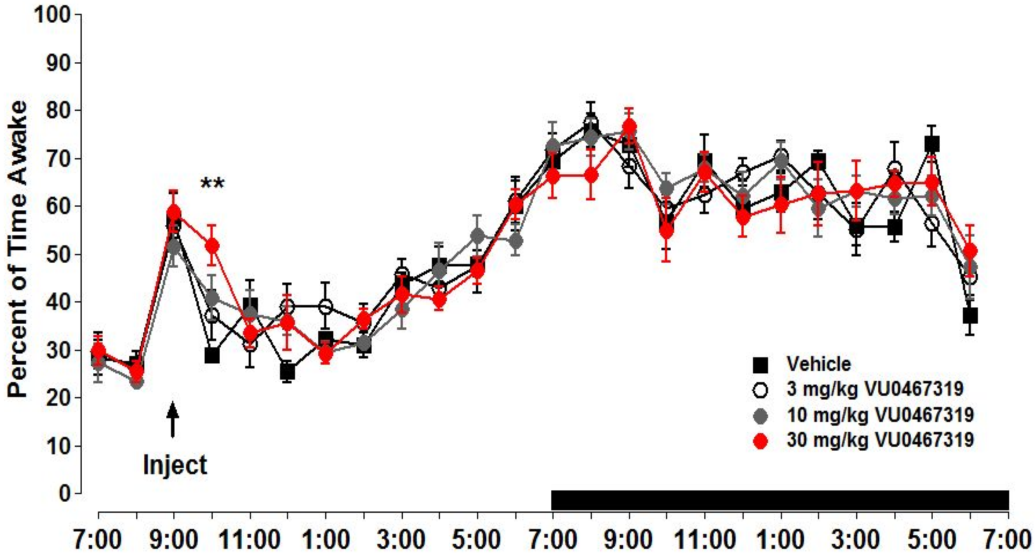

Figure S8. VU319 (PO) produced an increase in wakefulness in rats 1-2 hours post-administration in rats.

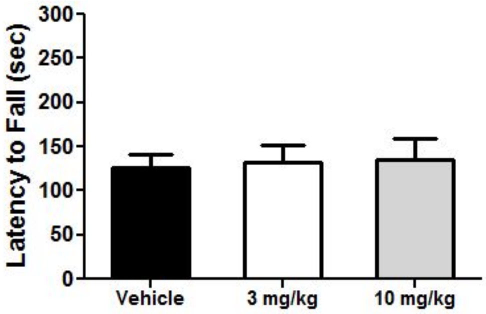

Figure S9. VU319 (PO) had no effect on the latency to fall from the accelerated rotarod in rats.

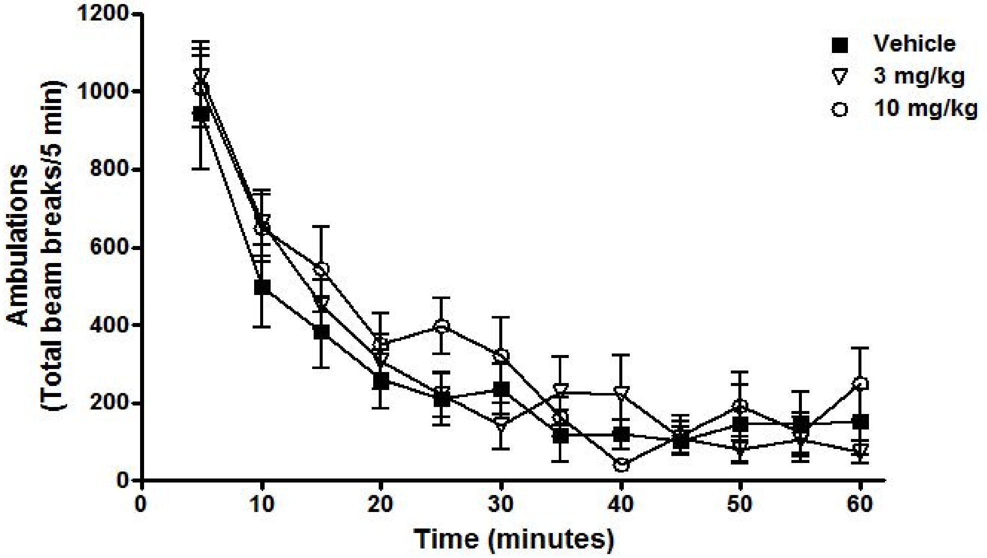

Figure S10. VU319 (PO) had no effect on basal locomotor activity in rats.

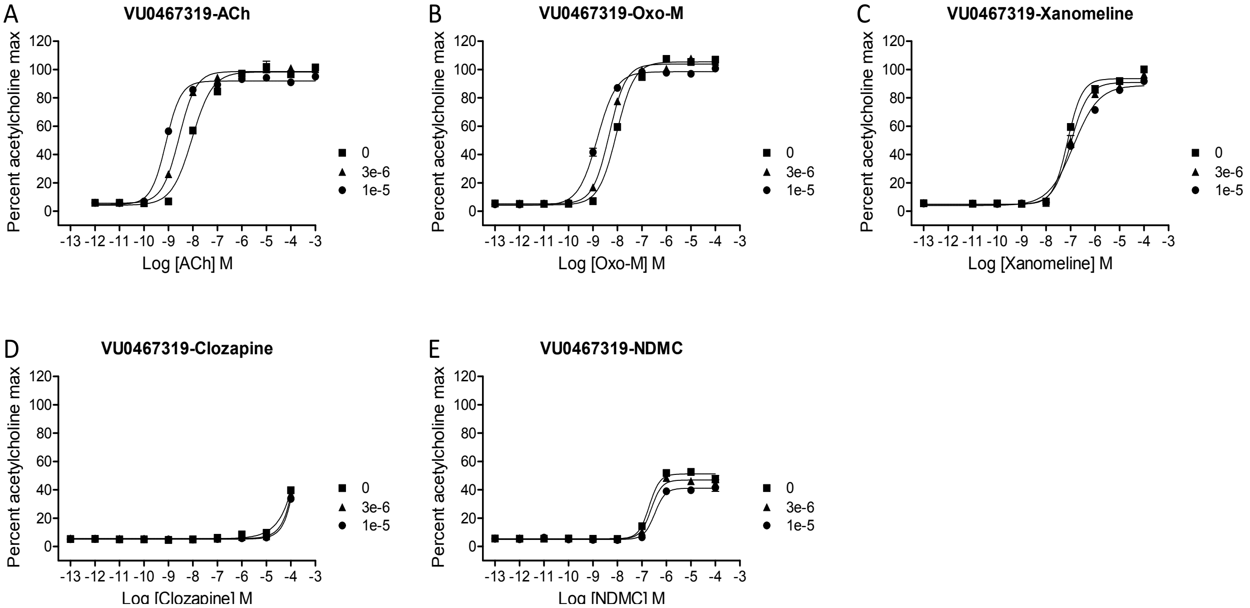

Figure S11. Efficacy experiments performed with VU319 in the presence of various orthosteric agonists. Vehicle control, 3 and 10 μM VU319 were incubated with cells expressing rat M_1 and then increasing concentrations of each agonist were added.

	Acetylcholine					Oxotremorine-M					Xanomeline				
	Mean EC_{50}	SEM	Fold shift	SEM	N	Mean EC_{50}	SEM	Fold shift	SEM	N	Mean EC_{50}	SEM	Fold shift	SEM	N
DMSO	9.40E-09	5.34E-10	1.0		2	9.18E-09	2.24E-10	1.0		2	7.49E-08	6.00E-10	1.0		2
3 uM	2.72E-09	7.50E-13	3.5	0.3	2	4.64E-09	2.69E-10	2.0	0.1	2	1.01E-07	2.43E-08	0.8	0.1	2
10 uM	7.90E-10	7.00E-11	11.9	0.7	2	1.51E-09	9.85E-11	6.1	0.3	2	1.33E-07	1.49E-08	0.6	0.1	2
	Clozapine					NDMC									
	Mean EC_{50}	SEM	Fold shift	SEM	N	Mean EC_{50}	SEM	Fold shift	SEM	N					
DMSO	No fit				2	1.97E-07	2.54E-07	1.0		2					
3 uM	No fit				2	2.36E-07	2.58E-07	0.8	0.0	2					
10 uM	No fit				2	3.30E-07	3.61E-07	0.6	0.0	2					

Table S2. Test of the ability of VU319 to shift the concentration-response curves of various agonists at the rat M_1 receptor.

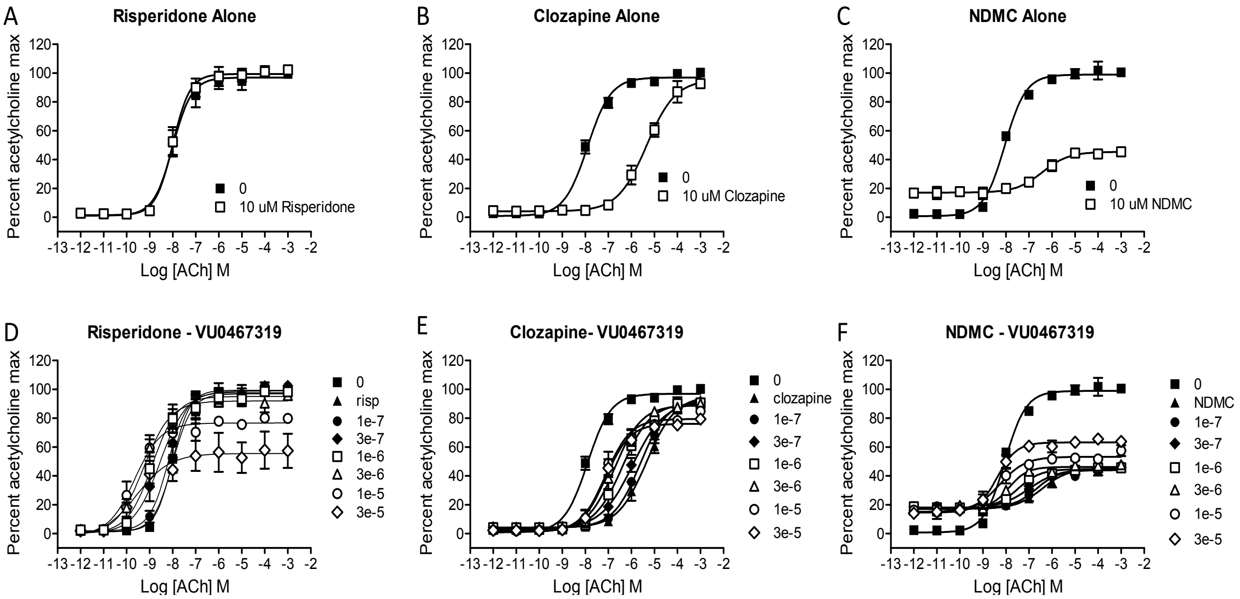

Figure S12. Exploration of the ability of VU319 to potentiate rat M_1 in vitro in the presence of risperidone, clozapine, or the major metabolite of clozapine, NDMC. In A, B, and C, effects of each compound on an ACh concentration-response curve are shown. Clozapine and NDMC shift the ACh response to the right. In D, E and F, the ability of VU319 to potentiate M_1 responses in the presence of these agents is shown. Data represent two-three independent experiments. performed in triplicate.

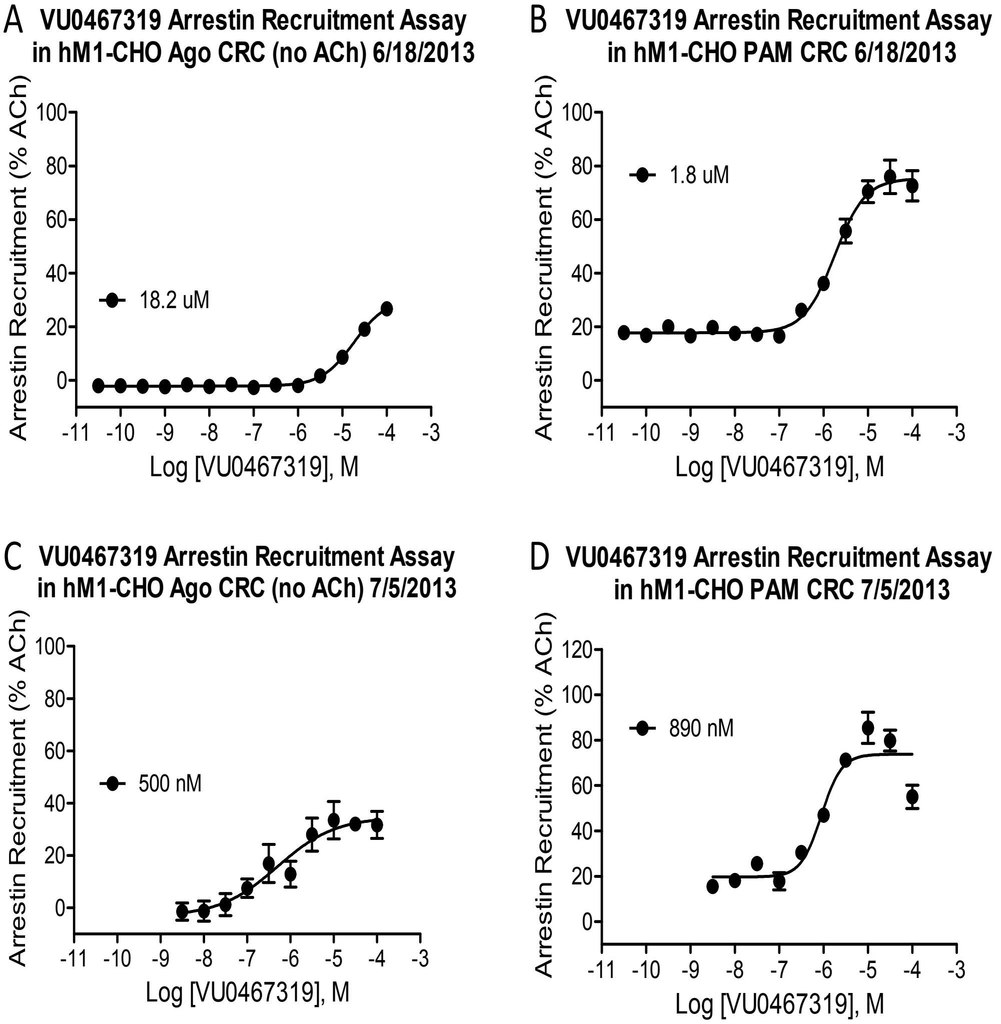

Figure S13. VU319 recruits β-arrestin 2 to the human M_1 receptor. Experiments were performed twice in quadruplicate. Values shown in each inset are the EC_{50} values for each experiment.

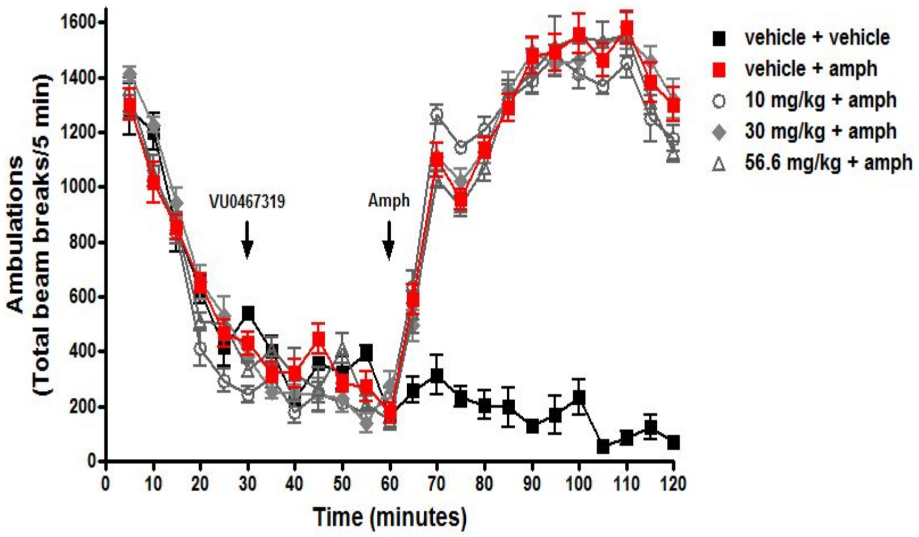

Figure S14. VU319 (PO) had no effect on reversing amphetamine-induced hyperactivity in rats.

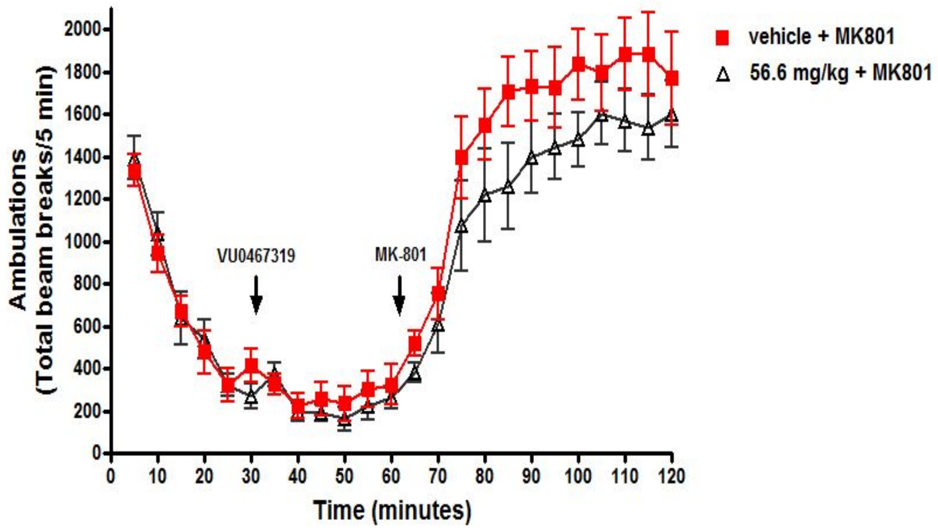

Figure S15. VU319 (PO) had no effect on reversing MK-801-induced hyperactivity in rats.

References

1. Suslick, K. S.; Doktycz, S. J. The sonochemistry of Zn powder. *J. Am. Chem. Soc.*, **1989**, 111 (6), 2342-2344. DOI: 10.1021/ja00188a081

2. Lie Ken Jie, M. S.; Mustafa J.; Pasha, M. K. An efficient ultrasound-assisted zinc reduction of fatty esters containing conjugated enynol and conjugated enynone systems *Lipids*, **1998**, 33 (9), 941-945. DOI: 10.1007/s11745-998-0291-x
